# An explainable machine learning approach to predicting carbapenem resistance in *Klebsiella pneumoniae* with imbalanced data and resistance-ratio validation

**DOI:** 10.3389/fcimb.2026.1906306

**Published:** 2026-09-16

**Authors:** Lili Geng, Yan He, Guangfei Yang, Shuang Zheng, Lingling Zhao, Qi Wang

**Affiliations:** 1 Dalian Medical University, Dalian, China; 2 Central Hospital of Dalian University of Technology (Dalian Municipal Central Hospital), Dalian, China; 3 Institute of Systems Engineering, Dalian University of Technology, Dalian, China; 4 Renmin University of China, Beijing, China; 5 The Second Hospital, Dalian Medical University, Dalian, China

**Keywords:** carbapenem-resistant Klebsiella pneumoniae, data imbalance, drug resistance, electronic health records, Klebsiella pneumoniae, machine learning, Shapley Additive Explanations (SHAP)

## Abstract

**Background:**

Carbapenem-resistant *Klebsiella pneumoniae* (CRKP) is a major global health threat. Predicting resistance from electronic medical records (EMRs) may support infection control and antimicrobial stewardship, but class imbalance can impair detection of resistant isolates. This study developed an interpretable machine-learning framework for predicting carbapenem-resistant versus carbapenem-susceptible *K. pneumoniae*.

**Methods:**

We retrospectively analyzed 5,794 records collected from 2014 to 2024, including 999 carbapenem-resistant and 4,795 carbapenem-susceptible isolates. The prediction anchor was culture-specimen collection (T0), and all predictors were restricted to information available at or before T0. Seven algorithms and 18 sampling strategies were evaluated using patient-grouped five-fold cross-validation with fold-specific preprocessing. The selected resampling pipeline was compared with Platt-calibrated XGBoost using sensitivity-targeted, F2-maximizing, and cost-sensitive thresholds. Feature-restriction analyses, multiplicity-adjusted comparisons, ICU subgroup analysis, reconstructed prevalence scenarios, calibration assessment, and same-center temporal validation were also performed.

**Results:**

XGBoost and LightGBM showed comparable discrimination after multiplicity adjustment. ENN–BLSMOTE–XGBoost increased sensitivity and reduced the very-major-error rate (VME) at the default threshold, but slightly reduced ranking discrimination and increased false-positive alerts. Sensitivity-targeted threshold optimization of calibrated XGBoost produced an operating point close to that of the resampled model, indicating that much of the sensitivity gain could be achieved without changing the training distribution. After all duration-based predictors were excluded, ENN–BLSMOTE–XGBoost retained an ROC AUC of 0.940 and a PR AUC of 0.833. In the 2025 temporal cohort, the calibrated ENN–BLSMOTE–XGBoost model achieved an ROC AUC of 0.889, a PR AUC of 0.684, a sensitivity of 0.749, and a VME of 0.251; Platt scaling reduced its Brier score from 0.121 to 0.108. SHAP analysis identified ICU admission, vascular system disease, days of indwelling urinary catheterization, days of carbapenem use, and days of endotracheal intubation as the five highest-attribution predictors. A web calculator was developed to provide calibrated probability estimates referenced to the 17.24% resistance prevalence of the development cohort.

**Conclusion:**

The proposed model effectively addresses class imbalance in predicting carbapenem resistance in *K. pneumoniae* and may support early identification of patients at risk of harboring carbapenem-resistant isolates, thereby informing infection control and antimicrobial stewardship.

## 1 Introduction

Antibiotic resistance is a major global public health challenge (Lv et al., 2025), with *Klebsiella pneumoniae* identified by the World Health Organization as a priority drug-resistant pathogen requiring new antimicrobial development (World Health Organization, 2017). Carbapenems, a first-line therapy for this common pathogen, are facing rising resistance to carbapenem-resistant Enterobacterales (CRE), threatening patient prognosis. Additionally, delayed identification can lead to nosocomial transmission, imposing a significant burden on clinical practice (Ting et al., 2025; Vásquez-Ponce et al., 2025). Accurate prediction of carbapenem resistance is critical for optimizing treatment regimens, improving cure rates, reducing healthcare costs, and curbing the spread of resistant strains.

With the advancement of medical informatization, electronic medical records (EMRs) accumulate rich patient data, including demographics, diagnoses, procedures, and medications (Manabe et al., 2021). Traditional resistance detection via culture and antimicrobial susceptibility testing (AST) is time-consuming, while machine learning offers efficient alternatives (Guliaev et al., 2025), enabling pattern extraction from complex data to model associations between patient characteristics and resistance (Yang et al., 2024). Previous studies have validated this potential: Sullivan et al. (2018) developed a logistic regression model for carbapenem-resistant *K. pneumoniae* bacteremia prediction (area under the receiver operating characteristic curve [auROC]=0.731); McGuire et al. (2021) achieved auROC=0.846 using XGBoost on EMR data; Salmi et al. (2024) reported auROC of 0.80–0.88 with bacterial type-specific models.

However, carbapenem resistance data are inherently imbalanced, with far fewer resistant cases than susceptible ones, leading to model bias toward the majority class and compromised minority class accuracy (Polat, 2018; Liu et al., 2019; Holmes et al., 2020; Li et al., 2023). This is clinically critical, as misclassifying resistant cases may delay effective treatment and exacerbate resistance spread (Li et al., 2022). In this context of low resistance prevalence, greater priority should be given to sensitivity (recall) for the resistant class. Therefore, addressing the data imbalance issue is particularly important. To address such class imbalance in medical data classification, researchers frequently rely on sampling strategies that adjust data distribution to mitigate model bias toward the majority class (Wang et al., 2021; Muksimova et al., 2024; Prithula et al., 2024). These methods are primarily categorized into three types: oversampling (e.g., Synthetic Minority Oversampling Technique [SMOTE] and its variants Borderline-SMOTE, Adaptive Synthetic Sampling [ADASYN]) that generate synthetic samples to enrich minority class representation (Silveira et al., 2022; Hassoun et al., 2023; Wu et al., 2024; Zhu et al., 2024; Li et al., 2025; Mastour et al., 2025); undersampling (e.g., Edited Nearest Neighbors [ENN] and Tomek Links [TL]) that remove redundant or noisy majority class samples (Chatterjee et al., 2023; Wei and Shi, 2025); and hybrid sampling (e.g., SMOTE-ENN, Borderline-SMOTE [BLSMOTE]-TL) that integrate both approaches to balance class distribution and improve data quality (Araf et al., 2024; Houssein et al., 2025; Zhang et al., 2025; Zhou et al., 2025). These strategies have been widely validated in medical informatics (Jeffrey et al., 2020; Wang et al., 2021; Yu et al., 2024). Beyond resampling, *post-hoc* probability calibration and clinically informed threshold optimization can improve probability accuracy and shift the operating point without altering the training distribution. In related work, Bonazzetti et al. developed ResPredAI, an EMR-based machine-learning framework for predicting resistance to four major antibiotic classes in patients with Gram-negative bloodstream infections, providing an adaptable workflow that accounts for local epidemiological and clinical characteristics (Bonazzetti et al., 2025). Despite this, few studies have systematically compared diverse sampling approaches for Carbapenem-Resistant *Klebsiella pneumoniae* (CRKP) prediction. Additionally, most models are validated under fixed Carbapenem-Resistant to Carbapenem-Susceptible (CR: CS) ratios, limiting generalizability across regions with varying resistance prevalence, and the “black-box” nature of machine learning hinders clinical acceptance due to insufficient interpretability.

To address these gaps, this study aimed to establish an interpretable and internally evaluated machine-learning framework for predicting carbapenem-resistant versus carbapenem-susceptible *K. pneumoniae* using EMR data. First, we compared seven baseline machine-learning algorithms to identify an appropriate reference model. Second, we systematically evaluated oversampling, undersampling, and hybrid-sampling strategies and benchmarked the selected resampling pipeline against Platt-calibrated XGBoost using sensitivity-constrained, F2-maximizing, cost-sensitive, and recall-targeted thresholds. Third, we assessed model performance through prediction-anchor feature-restriction analyses, same-center internal temporal evaluation, and reconstructed CR: CS prevalence scenarios, including calibration assessment and prevalence-prior correction. Fourth, we used SHapley Additive exPlanations and ICU subgroup analyses to characterize predictive associations. Finally, we developed a web-based calculator to provide calibrated probability estimates for antimicrobial-stewardship support. This work provides a scalable template for imbalanced medical data analysis and advances machine learning applications in antimicrobial resistance prediction. The flowchart of this study is illustrated in **Figure 1**.

**Figure 1:**
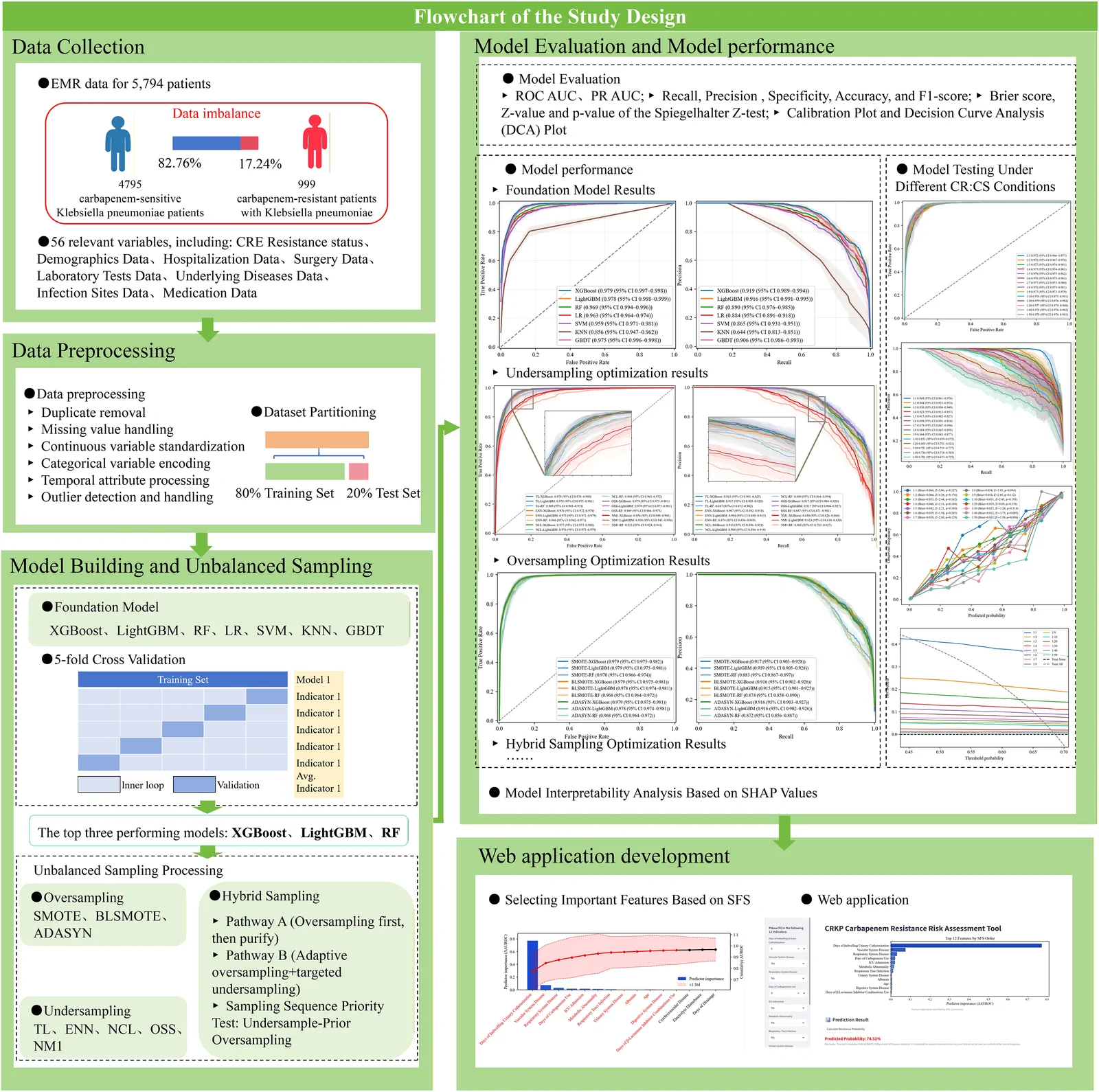
Flowchart of the study design. XGBoost, eXtreme Gradient Boosting; LightGBM, Light Gradient Boosting Machine; RF, Random Forest; LR, Logistic Regression; SVM, Support Vector Machine; KNN, K-Nearest Neighbors; GBDT, Gradient Boosting Decission Tree; SMOTE, Synthetic Minority Oversampling Technique; BLSMOTE, Borderline Synthetic Minority Oversampling Technique; ADASYN, Adaptive Synthetic Sampling; TL, Tomek Links; ENN, Edited Nearest Neighbors; NCL, Neighborhood Cleaning Rule; OSS, One-Sided Selection; NM1, Neighborhood Majority 1; CR, Carbapenem-Resistant; CS, Carbapenem-Susceptible; SHAP, SHapley Additive exPlanations; SFS, Sequential Forward Selection.

## 2 Methodology

### 2.1 Dataset

#### 2.1.1 Data source

This retrospective study collected EMRs of patients with culture-confirmed K. pneumoniae isolates at the Affiliated Central Hospital of Dalian University of Technology from 2014 to 2024, retrieving 8,816 initial records. The study was approved by the hospital’s Ethics Committee (Approval No.: YN2024-133-04), with informed consent waived in accordance with retrospective study guidelines and institutional ethical requirements.

#### 2.1.2 Data description

Guided by existing literature (Munoz-Price et al., 2013), EMR data were categorized into four dimensions: (1) Demographics, including age and gender; (2) Clinical data, including surgical records, medical orders, laboratory results, and comorbidities; (3) medication data, including antimicrobial use antimicrobial exposure during the 90-day look-back period ending at the culture-specimen collection anchor (T0); and (4) AST data, including carbapenem susceptibility results.

#### 2.1.3 Prediction anchor and intended clinical use

The predictor-information cutoff (T0) was defined as the time of culture-specimen collection (Li et al., 2014). The model was intended to be invoked after rapid species-level identification of the isolate as *Klebsiella pneumoniae* but before phenotypic AST results became available. Thus, the intended application was early reassessment of ongoing empirical antimicrobial therapy and antimicrobial-stewardship review rather than initial autonomous prescribing or replacement of AST.

All predictors were derived exclusively from information available at or before T0. Antimicrobial exposure was assessed during the 90-day look-back period ending at T0 (Lewin-Epstein et al., 2021; Jian et al., 2024). For antimicrobial treatments initiated during the current hospitalization, exposure duration was accumulated only through T0. Invasive-device durations were calculated from device insertion to T0, and laboratory predictors were defined as the most recent measurements available at or before T0. ICU status, procedures, diagnoses, comorbidities, and other clinical-state variables were included only when documented at or before T0. Although the model was invoked after species identification, information generated between specimen collection and model invocation was not incorporated.

#### 2.1.4 Outcome definition

Antimicrobial susceptibility interpretations were obtained from the laboratory information system and were based on the Clinical and Laboratory Standards Institute (CLSI) M100 edition implemented by the microbiology laboratory in the corresponding calendar year, ranging from M100-S24 in 2014 to M100, 34th edition, in 2024.

The carbapenem-resistance outcome was defined using phenotypic AST results for meropenem, imipenem, and ertapenem. An isolate was classified as carbapenem resistant if at least one tested target carbapenem was interpreted as resistant. An isolate was classified as carbapenem susceptible only when all available target-carbapenem results were interpreted as susceptible and no resistant or intermediate interpretation was present. Intermediate results were not grouped with either the resistant or susceptible category. Isolates with one or more intermediate interpretations but no resistant result, isolates without an interpretable result for any target carbapenem, and isolates without a definitive susceptibility outcome were excluded from the primary binary analysis.

#### 2.1.5 Data preprocessing and statistical analysis

Data preprocessing was performed to ensure data quality, completeness, and consistency. The procedures were as follows:

1. Duplicate removal: Exact duplicate records and repeated records from the same patient were removed. When multiple eligible records were available for one patient, only the record containing the most complete clinical, medication, laboratory, and microbiological information was retained.
2. Outcome exclusion and missing-data handling: Records without a definitive carbapenem-susceptibility outcome were excluded and were not imputed. Missingness was limited to three continuous laboratory predictors: absolute lymphocyte count, 92 of 5,794 records (1.59%); serum albumin, 58 of 5,794 records (1.00%); and platelet count, 91 of 5,794 records (1.57%). No missing values were present in the remaining predictors or in the outcome. The three continuous variables were imputed using medians estimated exclusively from the corresponding cross-validation training fold. A separate missingness indicator was added for each variable to preserve potentially informative test-ordering patterns.
3. Categorical-variable encoding: Binary clinical predictors, including sex, comorbidities, infection categories, procedures, invasive-device variables, antimicrobial-exposure variables, and ICU status, were represented as separate 0/1 indicators and contained no missing values; therefore, no median imputation was applied to these variables.
4. Outlier handling and standardization: Continuous predictors were winsorized at the 1st and 99th percentiles and standardized using Z-score transformation. The percentile thresholds, means, and standard deviations were estimated exclusively from the corresponding training fold.
5. Fold-specific preprocessing: In each cross-validation iteration, the training and held-out validation folds were defined before any data-dependent preprocessing was performed. Imputation values, missingness indicators, winsorization thresholds, standardization parameters, and encoding mappings were fitted using the training fold only and were subsequently applied unchanged to the held-out validation fold. The validation data were not used to estimate any preprocessing parameter.
6. Resampling: ENN, SMOTE, Borderline-SMOTE, and other resampling procedures were applied only after fold-specific preprocessing and exclusively to the training fold. No undersampling or synthetic oversampling was applied to the held-out validation fold, which retained the natural CR-to-CS distribution for performance evaluation.

The final preprocessed dataset is shown in **Supplementary Table 1**. It included 5,794 independent samples, comprising 999 CR cases and 4,795 CS cases. After excluding irrelevant features based on literature and medical expertise, 56 candidate features were retained for modeling. Categorical variables are presented as counts and percentages, and P-values were calculated using the chi-square test, while continuous variables are reported as medians (interquartile ranges) with P-values calculated via the Mann–Whitney U test.

### 2.2 Models

#### 2.2.1 Baseline machine learning models

Seven classical algorithms were selected as baselines for CRKP prediction: Logistic Regression, Support Vector Machine, K-Nearest Neighbors (KNN), Random Forest (RF), Gradient Boosting Decision Tree, eXtreme Gradient Boosting (XGBoost), and Light Gradient Boosting Machine (LightGBM). All models were evaluated using five-fold cross-validation. In each fold, models were trained on four folds and evaluated on the held-out fold. Sampling procedures were applied only to the training folds to avoid data leakage. Hyperparameters are detailed in **Supplementary Table 2**, with implementation via Python 3.9 using the scikit-learn, XGBoost, and LightGBM libraries to ensure reproducibility.

#### 2.2.2 Model evaluation and statistical tests

Considering the clinical characteristics of carbapenem resistance prediction, a comprehensive evaluation was performed using metrics covering four core dimensions: discriminative ability, classification performance, calibration, and clinical utility.

1. Discriminative ability: The Area Under the Receiver Operating Characteristic Curve (ROC AUC) was used to assess the overall ability of models to distinguish between carbapenem-resistant and carbapenem-susceptible *K. pneumoniae* cases. Given the data imbalance, the Area Under the Precision-Recall Curve (PR AUC) was additionally used to characterize performance in identifying the minority class of carbapenem-resistant cases.
2. Threshold-dependent classification performance. For the primary model-development comparisons, classification performance was summarized using recall (sensitivity), precision (positive predictive value), specificity, accuracy, and F1-score. The formulas for these metrics are as shown in (Equations 1–5):

(1) $$\text{Recall}=\frac{\text{TP}}{\text{TP}+\text{FN}}$$

(2) $$\text{Precision}=\frac{\text{TP}}{\text{TP}+\text{FP}}$$

(3) $$\text{Specificity}=\frac{\text{TN}}{\text{TN}+\text{FP}}$$

(4) $$\text{Accuracy }=\frac{\text{TP }+\text{ TN}}{\text{TP }+\text{ TN }+\text{ FP }+\text{ FN}}$$

(5) $$\text{F}1=\frac{2\times \text{Recall}\times \text{Precision}}{\text{Recall}+\text{Precision}}$$

For analyses explicitly addressing asymmetric clinical error costs—including threshold optimization, comparison between threshold adjustment and resampling, ICU subgroup evaluation, and internal temporal validation—we additionally reported the F2-score, very-major-error rate (VME), major-error rate (ME), and confusion matrices. The formulas for these metrics are as shown in (Equations 6–8):

(6) $$\text{F}2=\frac{5\times \text{Precision}\times \text{Recall}}{4\times \text{Precision}+\text{Recall}}$$

(7) $$\text{VME}=\frac{\text{FN}}{\text{TP}+\text{FN}}$$

(8) $$\text{ME}=\frac{\text{FP}}{\text{FP}+\text{TN}}$$

The F2-score was included because missing a resistant record was considered more consequential than generating a false-positive resistance alert.

3. Probability calibration. The Brier score was used to quantify the mean squared difference between predicted probabilities and observed outcomes. For the baseline XGBoost pipeline, the selected resampling pipeline, and their *post-hoc* calibrated versions, calibration was further assessed using calibration curves, calibration intercept, calibration slope, expected calibration error based on 10 bins (ECE), and the Spiegelhalter Z-test. Ideal calibration corresponds to a calibration intercept of 0 and a calibration slope of 1. The Spiegelhalter test was interpreted together with the Brier score, calibration curve, intercept, and slope rather than as a stand-alone measure of calibration quality.

4. Clinical utility. Decision curve analysis was performed for the final deployment-oriented model pipelines to estimate clinical net benefit across a range of threshold probabilities. DCA was not repeated for every exploratory sampling strategy or sensitivity analysis.

Raw probability outputs were initially evaluated at the conventional threshold of 0.50. To benchmark resampling against a non-resampling alternative, unsampled XGBoost was *post-hoc* calibrated using Platt scaling and evaluated at several clinically oriented operating thresholds. These included a sensitivity-targeted threshold selected to achieve sensitivity of at least 0.85 in the training data, an F2-maximizing threshold, and exploratory cost-sensitive thresholds assigning VME: ME relative costs of 2:1, 5:1, and 10:1.

During model development, performance was estimated using five-fold patient-grouped cross-validation. All preprocessing, resampling, model fitting, probability calibration, and threshold selection were performed using the training portion of each fold only. Pooled out-of-fold predictions were used for performance estimation and probability recalibration. Ninety-five percent confidence intervals for key metrics and paired between-model differences were estimated using 1,000 patient-cluster bootstrap resamples.

To reduce the risk of false-positive inference arising from the evaluation of multiple algorithms, sampling strategies, and performance metrics, formal statistical testing was restricted to prespecified comparison families rather than all possible pairwise model contrasts. These included comparisons between XGBoost and LightGBM for reference-model selection, comparisons between the selected ENN–BLSMOTE–XGBoost pipeline and unsampled XGBoost, and a separate comparison family for the ICU subgroup analysis. Within each family, p-values were adjusted using both the Benjamini–Hochberg false-discovery-rate procedure and the Bonferroni procedure. The full screening results for the remaining models and sampling strategies were interpreted descriptively. Statistical significance was defined as a two-sided adjusted p-value below 0.05.

#### 2.2.3 Oversampling for imbalanced data

To address the class imbalance, where the CR: CS ratio was approximately 1:4.8, and to mitigate bias toward the majority class CS, three mainstream oversampling techniques were used to enhance the representation of the minority class CR. These techniques included SMOTE, BLSMOTE, and ADASYN. Oversampling was performed only after the training and validation sets had been separated, and it was applied exclusively to the training folds during cross-validation to avoid data leakage.

#### 2.2.4 Undersampling for imbalanced data

Five undersampling methods were used to optimize the distribution of the majority class CS by removing redundant or noisy samples while preserving key clinical information. These methods included TL, ENN, Neighborhood Cleaning Rule (NCL), One-Sided Selection (OSS), and Neighborhood Majority 1 (NM1). All CR samples were retained, with undersampling limited to cross-validation training sets for subsequent model training.

#### 2.2.5 Hybrid sampling for imbalanced data

Two hybrid pathways were designed to combine the advantages of oversampling and undersampling. Additional reversed-order strategies were also included to explore the effect of sampling order. Pathway A followed an oversampling-first and then purification strategy. The combinations included SMOTE-TL, SMOTE-ENN, BLSMOTE-ENN, and BLSMOTE-TL. Pathway B combined adaptive oversampling with targeted undersampling. The combinations included ADASYN-TL, SMOTE-NCL, SMOTE-NM1, and ADASYN-NM1. Reversed-order strategies, including ENN-BLSMOTE and NM1-SMOTE, prioritized majority class purification before oversampling. Parameters were kept consistent with those described in Sections 2.2.3 and 2.2.4 to ensure comparability.

#### 2.2.6 Model interpretability based on SHAP values

SHAP was used to provide global and local model interpretability by quantifying the contribution of each feature to the model output. Mean absolute SHAP values were used to summarize global feature attribution, whereas SHAP summary and waterfall plots were used to characterize the direction and magnitude of feature contributions at the population and individual levels. Because SHAP values explain model predictions rather than causal mechanisms, all SHAP findings were interpreted as predictive associations and not as estimates of causal effects.

#### 2.2.7 Model testing under varying CR: CS ratios

Given regional variations in clinical carbapenem resistance rates, with CR: CS ratios potentially ranging from 1:1 to 1:50 across different hospitals or regions, the model’s robustness under different imbalance degrees was verified. Fourteen CR: CS ratio gradients (1:1, 1:2, 1:3, 1:4, 1:5, 1:6, 1:7, 1:8, 1:9, 1:10, 1:20, 1:30, 1:40, 1:50) were constructed via stratified sampling to simulate data distributions in different clinical scenarios.

#### 2.2.8 Web application

A free web calculator (https://crkp-calculator-baxwok2n638p2knfa6fkxb.streamlit.app/) was developed using the optimal model and 12 core features identified via Sequential Forward Selection (SFS). Built with Python (Python 3) Streamlit framework, it provides structured data entry, real-time resistance probability outputs, and feature-importance visualization to support clinical interpretability and translation.

## 3 Results

### 3.1 Performance of baseline machine learning models

To evaluate baseline machine learning models for predicting carbapenem resistance in *K. pneumoniae*, seven classical algorithms (XGBoost, LightGBM, RF, Logistic Regression, Support Vector Machine, KNN, Gradient Boosting Decision Tree) were compared via five-fold cross-validation, with performance metrics summarized in **Table 1** and visualized in **Figure 2**. The hyperparameter settings of these baseline models are provided in **Supplementary Table 2**.

**Table 1:** Performance metrics of baseline machine learning models (5-fold cross-validation).

Model	ROC AUC	PR AUC	Recall	Precision	Specificity	Accuracy	F1	Brier score	Spiegelhalter Z	Spiegelhalter p
XGBoost	0.978 (95% CI 0.975–0.981)	0.916 (95% CI 0.904–0.928)	0.730	0.879	0.979	0.936	0.797	0.044	2.804	0.225
LightGBM	0.978 (95% CI 0.974–0.981)	0.916 (95% CI 0.903–0.928)	0.725	0.887	0.981	0.936	0.797	0.047	9.932	0.000
RF	0.969 (95% CI 0.965–0.974)	0.891 (95% CI 0.875–0.906)	0.538	0.950	0.994	0.916	0.686	0.059	-10.528	0.000
LR	0.962 (95% CI 0.956–0.968)	0.884 (95% CI 0.867–0.899)	0.684	0.864	0.970	0.931	0.788	0.050	1.560	0.168
SVM	0.958 (95% CI 0.952–0.964)	0.868 (95% CI 0.850–0.884)	0.690	0.861	0.977	0.927	0.766	0.055	0.703	0.338
KNN	0.861 (95% CI 0.847–0.874)	0.652 (95% CI 0.624–0.679)	0.412	0.836	0.983	0.885	0.549	0.087	16.478	0.000
GBDT	0.973 (95% CI 0.969–0.977)	0.901 (95% CI 0.887–0.914)	0.712	0.875	0.979	0.933	0.785	0.048	5.819	0.002

**Figure 2:**
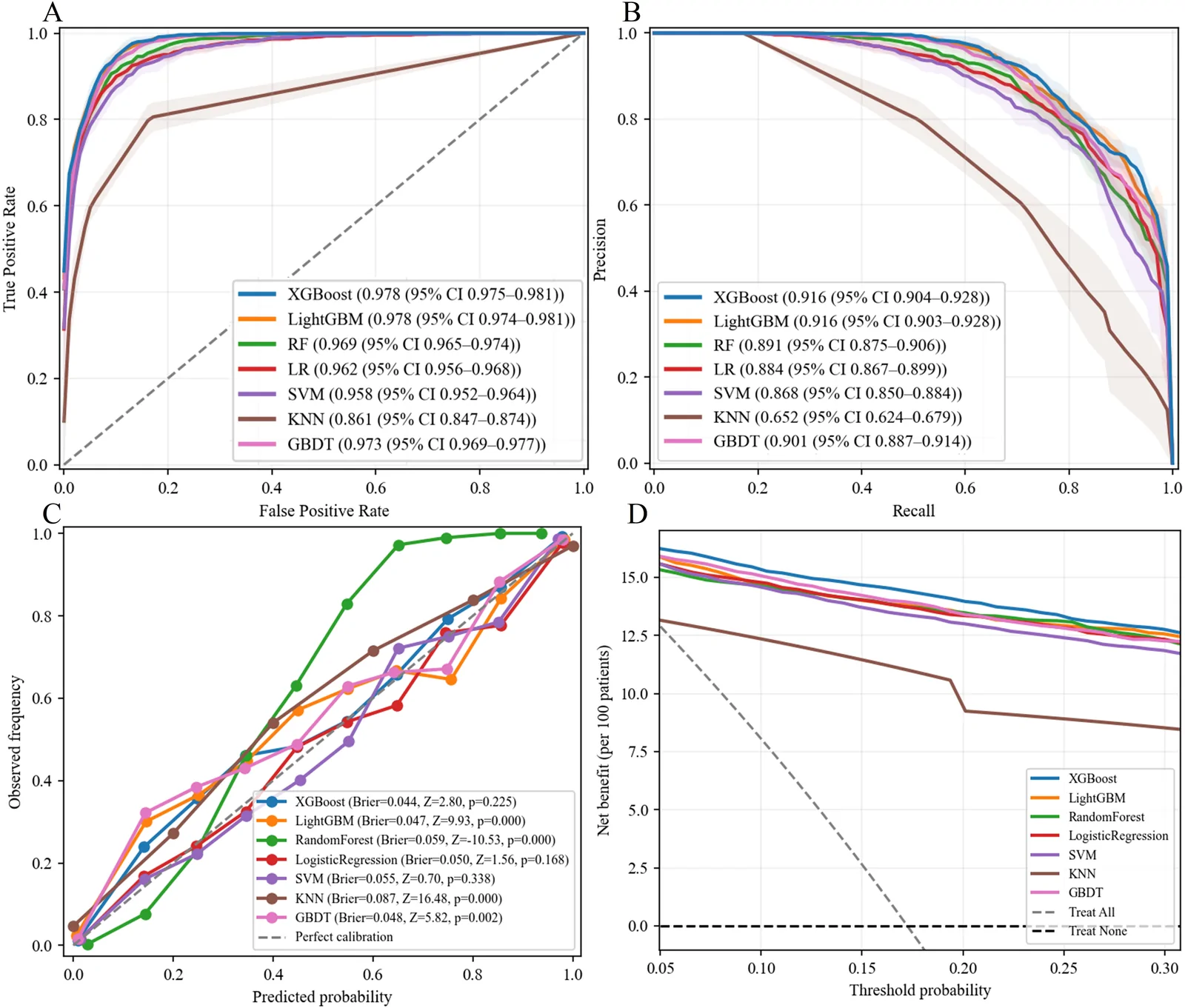
Performance visualization of baseline machine learning models. **(A)** Receiver Operating Characteristic (ROC) Curves (with Area Under the Curve (AUC) and 95% Confidence Interval (CI)); **(B)** Precision-Recall (PR) Curves (with PR AUC and 95% CI); **(C)** Calibration Curves (with Brier Score and Spiegelhalter’s Test Results); **(D)** Decision Curve Analysis (DCA) for clinical net benefit assessment. XGBoost, eXtreme Gradient Boosting; LightGBM, Light Gradient Boosting Machine; RF, Random Forest; LR, Logistic Regression; SVM, Support Vector Machine; KNN, K-Nearest Neighbors; GBDT, Gradient Boosting Decision Tree.

As shown in **Table 1**, all models except KNN achieved ROC AUC > 0.95, with XGBoost (0.978, 95% CI: 0.975–0.981) and LightGBM (0.978, 95% CI: 0.974–0.981) leading in discriminative ability (ROC AUC/PR AUC). XGBoost balanced recall (0.730), precision (0.879), and calibration (Brier=0.044, Spiegelhalter p=0.225), and outperformed extreme clinical strategies via DCA (0.05–0.30 threshold). RF exhibited high specificity but low recall, while KNN performed poorly across metrics. XGBoost and LightGBM demonstrated comparable discrimination, with paired multiplicity-adjusted comparisons showing no significant difference in ROC AUC or PR AUC. XGBoost was retained as the reference baseline because of its slightly lower Brier score and continuity with the subsequent SHAP, calibration, threshold-optimization, temporal-validation, and deployment analyses, rather than statistically superior discrimination. RF was retained as a structurally distinct bagging comparator.

### 3.2 Comparison and analysis of oversampling methods

To examine sampling effects across representative tree-based architectures, the primary experiments included XGBoost and LightGBM as the two strongest boosting baselines and RF as an algorithmically distinct bagging comparator. Each classifier was evaluated in combination with three commonly used oversampling techniques—SMOTE, BLSMOTE, and ADASYN. All models were validated via five-fold cross-validation, with performance metrics summarized in **Table 2** and visualized in **Figure 3**.

**Table 2:** Performance of oversampling-optimized models (five-fold cross-validation).

Model	ROC AUC	PR AUC	Recall	Precision	Specificity	Accuracy	F1	Brier score	Spiegelhalter Z	Spiegelhalter p
SMOTE-XGBoost	0.979 (95% CI 0.975–0.982)	0.917 (95% CI 0.903–0.928)	0.775	0.845	0.970	0.937	0.808	0.044	2.138	0.310
SMOTE-LightGBM	0.979 (95% CI 0.975–0.981)	0.919 (95% CI 0.905–0.928)	0.759	0.859	0.974	0.937	0.806	0.045	8.160	0.000
SMOTE-RF	0.970 (95% CI 0.966–0.974)	0.883 (95% CI 0.867–0.897)	0.736	0.799	0.961	0.922	0.765	0.058	-14.818	0.000
BLSMOTE-XGBoost	0.979 (95% CI 0.975–0.981)	0.916 (95% CI 0.902–0.926)	0.775	0.834	0.968	0.934	0.803	0.044	2.489	0.257
BLSMOTE-LightGBM	0.978 (95% CI 0.974–0.981)	0.915 (95% CI 0.901–0.925)	0.760	0.861	0.974	0.937	0.807	0.046	8.646	0.000
BLSMOTE-RF	0.968 (95% CI 0.964–0.972)	0.874 (95% CI 0.858–0.890)	0.756	0.775	0.954	0.920	0.765	0.060	-14.373	0.000
ADASYN-XGBoost	0.979 (95% CI 0.975–0.981)	0.916 (95% CI 0.903–0.927)	0.776	0.838	0.969	0.935	0.806	0.044	2.276	0.264
ADASYN-LightGBM	0.978 (95% CI 0.974–0.981)	0.916 (95% CI 0.902–0.926)	0.761	0.862	0.974	0.938	0.808	0.046	8.270	0.000
ADASYN-RF	0.968 (95% CI 0.964–0.972)	0.872 (95% CI 0.856–0.887)	0.775	0.757	0.948	0.918	0.766	0.061	-14.438	0.000

**Figure 3:**
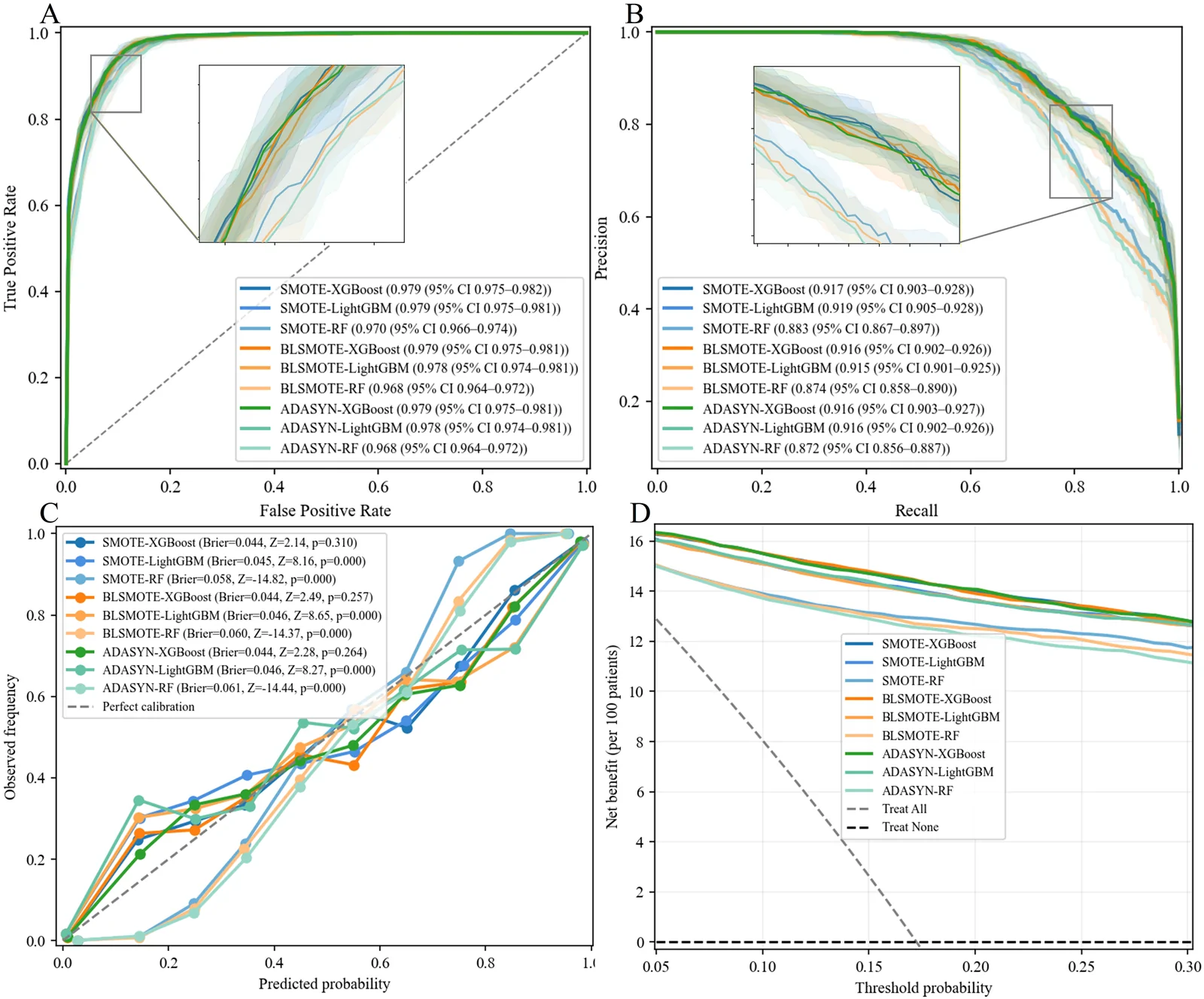
Performance visualization of oversampling-optimized models. **(A)** Receiver Operating Characteristic (ROC) Curves (with Area Under the Curve (AUC) and 95% Confidence Interval (CI)); **(B)** Precision-Recall (PR) Curves (with PR AUC and 95% CI); **(C)** Calibration Curves (with Brier Score and Spiegelhalter’s Test Results); **(D)** Decision Curve Analysis (DCA) for clinical net benefit assessment. SMOTE, Synthetic Minority Oversampling Technique; BLSMOTE, Borderline Synthetic Minority Oversampling Technique; ADASYN, Adaptive Synthetic Sampling; XGBoost, eXtreme Gradient Boosting; LightGBM, Light Gradient Boosting Machine; RF, Random Forest.

As shown in **Table 2**, all optimized models maintained ROC AUC > 0.968, with SMOTE-XGBoost, BLSMOTE-XGBoost, and ADASYN-XGBoost achieving the highest ROC AUC (0.979, 95% CI: 0.975–0.982) and PR AUC (0.916–0.917). SMOTE-XGBoost (recall=0.775, F1 = 0.808) and ADASYN-LightGBM (recall=0.761, F1 = 0.808) balanced classification performance, while RF-based models showed improved recall (0.736–0.775) but lower precision. XGBoost-based models retained optimal overall performance, with oversampling effectively enhancing minority class identification.

### 3.3 Comparison and analysis of undersampling methods

To comprehensively investigate the impact of sampling strategies on the imbalanced carbapenem resistance dataset, and considering that undersampling can yield superior optimization outcomes for certain imbalanced scenarios, we optimized three core models, XGBoost, LightGBM, and RF, using five undersampling techniques: TL, ENN, NCL, OSS, and NM1, resulting in 15 optimized models. All models were validated via five-fold cross-validation, with performance metrics summarized in **Table 3** and visualized in **Figure 4**.

**Table 3:** Performance of undersampling-optimized models (5-fold cross-validation).

Model	ROC AUC	PR AUC	Recall	Precision	Specificity	Accuracy	F1	Brier score	Spiegelhalter Z	Spiegelhalter p
TL-XGBoost	0.978 (95% CI 0.974–0.980)	0.915 (95% CI 0.901–0.925)	0.746	0.859	0.974	0.935	0.798	0.045	3.146	0.134
TL-LightGBM	0.978 (95% CI 0.975–0.981)	0.917 (95% CI 0.905–0.928)	0.738	0.880	0.979	0.937	0.802	0.046	9.487	0.000
TL-RF	0.969 (95% CI 0.965–0.973)	0.887 (95% CI 0.872–0.902)	0.553	0.934	0.992	0.916	0.695	0.059	-10.815	0.000
ENN-XGBoost	0.976 (95% CI 0.972–0.979)	0.907 (95% CI 0.892–0.918)	0.821	0.780	0.951	0.929	0.799	0.049	5.195	0.001
ENN-LightGBM	0.975 (95% CI 0.971–0.979)	0.904 (95% CI 0.889–0.915)	0.819	0.796	0.956	0.933	0.807	0.051	13.447	0.000
ENN-RF	0.966 (95% CI 0.962–0.971)	0.874 (95% CI 0.856–0.889)	0.673	0.831	0.971	0.920	0.743	0.058	-12.842	0.000
NCL-XGBoost	0.977 (95% CI 0.973–0.980)	0.910 (95% CI 0.896–0.921)	0.799	0.804	0.959	0.931	0.801	0.047	3.067	0.190
NCL-LightGBM	0.976 (95% CI 0.973–0.979)	0.908 (95% CI 0.894–0.919)	0.794	0.798	0.958	0.929	0.795	0.050	11.302	0.000
NCL-RF	0.968 (95% CI 0.963–0.972)	0.880 (95% CI 0.864–0.894)	0.638	0.868	0.980	0.921	0.735	0.058	-13.414	0.000
OSS-XGBoost	0.979 (95% CI 0.976–0.982)	0.918 (95% CI 0.905–0.929)	0.756	0.862	0.975	0.937	0.805	0.044	2.445	0.336
OSS-LightGBM	0.978 (95% CI 0.974–0.981)	0.916 (95% CI 0.903–0.927)	0.733	0.875	0.978	0.936	0.797	0.046	9.166	0.000
OSS-RF	0.969 (95% CI 0.965–0.973)	0.888 (95% CI 0.872–0.902)	0.545	0.946	0.994	0.916	0.691	0.059	-10.861	0.000
NM1-XGBoost	0.956 (95% CI 0.949–0.961)	0.850 (95% CI 0.826–0.864)	0.922	0.533	0.832	0.847	0.675	0.114	23.913	0.000
NM1-LightGBM	0.950 (95% CI 0.943–0.956)	0.833 (95% CI 0.810–0.850)	0.923	0.507	0.812	0.832	0.654	0.137	48.185	0.000
NM1-RF	0.933 (95% CI 0.924–0.941)	0.808 (95% CI 0.783–0.827)	0.899	0.464	0.783	0.803	0.612	0.132	-8.470	0.000

**Figure 4:**
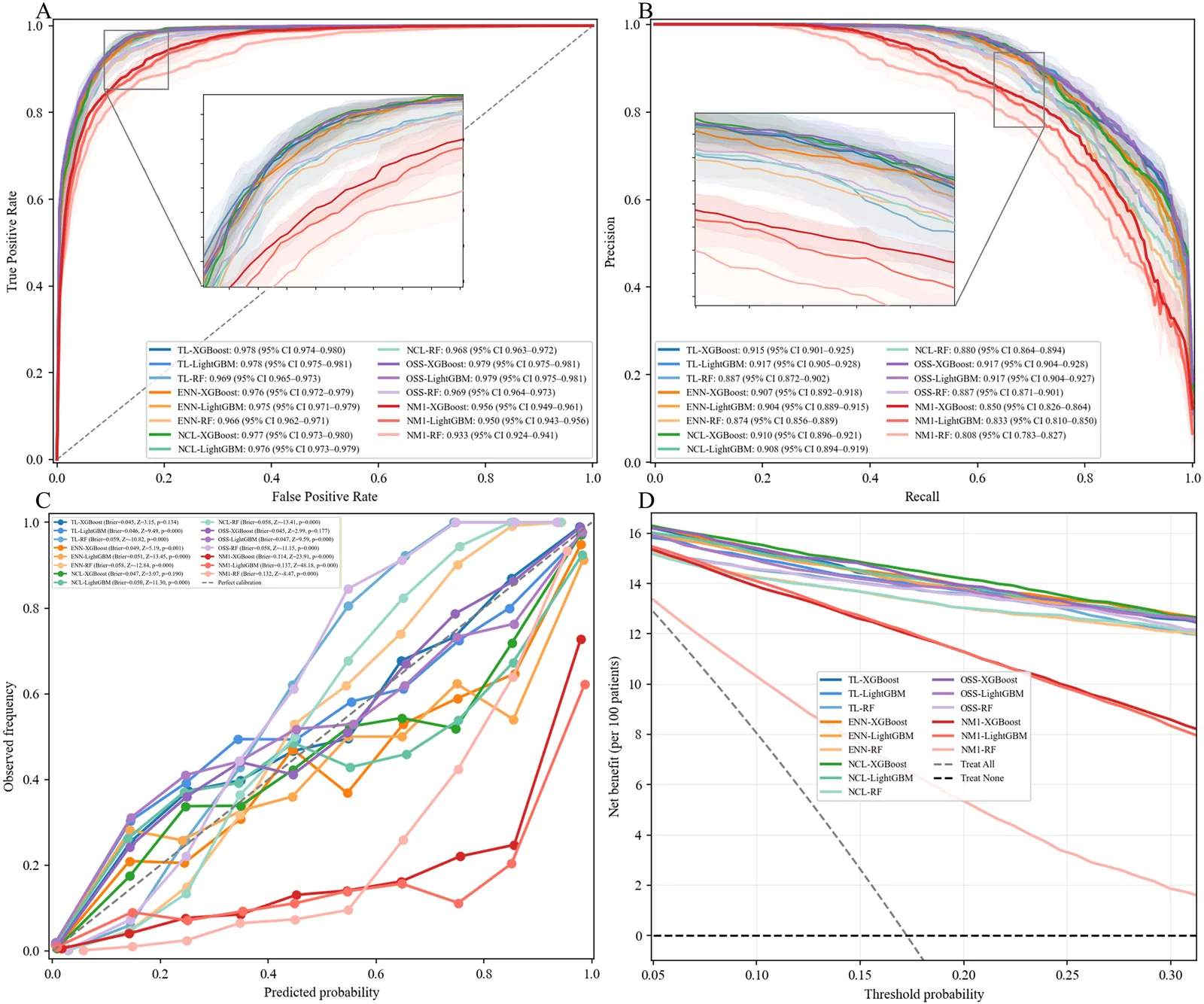
Performance visualization of undersampling-optimized models. **(A)** Receiver Operating Characteristic (ROC) Curves (with Area Under the Curve (AUC) and 95% Confidence Interval (CI)); **(B)** Precision-Recall (PR) Curves (with PR AUC and 95% CI); **(C)** Calibration Curves (with Brier Score and Spiegelhalter’s Test Results); **(D)** Decision Curve Analysis (DCA) for clinical net benefit assessment. TL, Tomek Links; ENN, Edited Nearest Neighbors; NCL, Neighborhood Cleaning Rule; OSS, One-Sided Selection; NM1, Neighborhood Majority 1; XGBoost, eXtreme Gradient Boosting; LightGBM, Light Gradient Boosting Machine; RF, Random Forest.

As shown in **Table 3**, most undersampled models maintained strong discriminative ability. ENN-optimized models achieved the highest recall versus baselines, while NM1-optimized models had extremely high recall but unacceptably low precision (0.533), risking over-classification of susceptible cases. Prioritizing recall for carbapenem resistance prediction, given the higher cost of missing resistant cases, ENN-XGBoost balanced high recall with acceptable precision (0.780), avoiding under-identification and over-treatment. Calibration curves (**Figure 4C**) confirmed OSS-XGBoost, NCL-XGBoost, and ENN-XGBoost were well-calibrated, and DCA (**Figure 4D**) showed ENN-, OSS-, TL-based XGBoost/LightGBM models outperformed extreme clinical strategies (0.05–0.30 threshold). Overall, undersampling effectively improved recall, with ENN-XGBoost emerging as the most clinically relevant model for further validation.

### 3.4 Comparison and analysis of hybrid sampling methods

Hybrid sampling integrates oversampling and undersampling to mitigate limitations of single strategies, oversampling-induced noise, and undersampling-related information loss. Following an “oversampling-first then undersampling” paradigm, we designed two pathways (Pathway A: SMOTE-TL, SMOTE-ENN, BLSMOTE-ENN, BLSMOTE-TL; Pathway B: ADASYN-TL, SMOTE-NCL, SMOTE-NM1, ADASYN-NM1) and reversed-order strategies (ENN-BLSMOTE, NM1-SMOTE) to explore the impact of sampling order. All 30 hybrid models were validated via five-fold cross-validation (**Supplementary Table 3**; **Supplementary Figures S1**, **S2**).

As shown in **Supplementary Table 3** and **Supplementary Figures S1**, **S2**, “oversampling-first” strategies performed comparably to single oversampling methods, while NM1-SMOTE mirrored NM1 undersampling, showing high recall and low precision. ENN-BLSMOTE-XGBoost (undersampling-first) exhibited synergistic effects: ENN reduced majority-class noise, and BLSMOTE enhanced minority-class boundary samples, achieving the highest recall (0.852) with acceptable precision (0.761). Accordingly, ENN–BLSMOTE–XGBoost was retained as the sensitivity-oriented candidate because it improved resistant-case detection and reduced VME while maintaining ME at a comparatively controlled level.

Because conventional GBDT also demonstrated strong baseline performance and belongs to the same gradient-boosting family as XGBoost and LightGBM, RF was retained in the primary sampling experiments to provide an algorithmically distinct bagging comparator. Complete oversampling, undersampling, and hybrid-sampling results for GBDT are reported in **Supplementary Table 4**. The GBDT extension showed a similar pattern. ENN–BLSMOTE–GBDT increased recall from 0.698 for unsampled GBDT to 0.838 and reduced VME from 0.302 to 0.162, while maintaining a substantially more balanced precision and error profile than the more aggressive NM1–SMOTE–GBDT strategy (precision, 0.732 vs 0.517; ME, 0.064 vs 0.178). Although NM1–SMOTE–GBDT achieved the highest recall (0.914) and the lowest VME (0.086), this gain was accompanied by markedly reduced specificity (0.822) and a substantially increased false-positive burden. These findings indicate that the sensitivity-oriented effect of ENN–BLSMOTE was broadly reproducible across gradient-boosting learners and offered a more balanced trade-off between reducing missed resistant cases and controlling ME.

### 3.5 Multiplicity-adjusted comparisons and model selection

After multiplicity adjustment, XGBoost and LightGBM showed comparable discrimination (**Supplementary Table 5**). For LightGBM versus XGBoost, the ROC AUC difference was −0.0004 (95% CI, −0.0016 to 0.0009; BH-adjusted p=0.527; Bonferroni-adjusted p=1.000), and the PR AUC difference was −0.0017 (95% CI, −0.0062 to 0.0028; BH-adjusted p=0.382; Bonferroni-adjusted p=1.000). At the default threshold of 0.50, XGBoost showed a slightly more favorable operating-point profile than LightGBM, with higher sensitivity, PPV, and specificity, together with lower VME, ME, and Brier score. Based on the study objective of minimizing VME while maintaining ME at an acceptable level, XGBoost was selected as the reference baseline for the subsequent sampling experiments.

Compared with unsampled XGBoost, ENN–BLSMOTE–XGBoost showed slightly lower ROC AUC (difference, −0.0027; 95% CI, −0.0046 to −0.0008; BH-adjusted p=0.030; Bonferroni-adjusted p=0.060) and PR AUC (difference, −0.0121; 95% CI, −0.0203 to −0.0047; BH-adjusted p=0.010; Bonferroni-adjusted p=0.020). However, at the default threshold of 0.50, ENN–BLSMOTE–XGBoost increased sensitivity by 0.108, increased the F2-score by 0.063, and reduced VME by 0.108 relative to XGBoost; these differences remained significant after multiplicity adjustment.

These results indicate complementary operating characteristics rather than uniform superiority. XGBoost provided better control of false-positive resistance alerts, while ENN–BLSMOTE–XGBoost more effectively reduced missed resistant cases. Because the prespecified clinical objective of this study was to minimize VME while maintaining ME at an operationally acceptable level, ENN–BLSMOTE–XGBoost was retained as the sensitivity-oriented candidate for early resistance screening and antimicrobial-stewardship review.

### 3.6 Threshold-optimization benchmark against the calibrated baseline

To assess whether the sensitivity improvement observed after resampling could also be achieved by adjusting the decision threshold of the unsampled model (Bonazzetti et al., 2025), several operating points of Platt-calibrated XGBoost were compared with ENN–BLSMOTE–XGBoost at the fixed threshold of 0.50 (**Table 4**).

**Table 4:** Operating-point comparison of platt-calibrated XGBoost and ENN–BLSMOTE–XGBoost.

Operating point	Threshold	Sensitivity	Precision	Specificity	F1-score	F2-score	VME	ME
Platt-calibrated XGBoost, fixed threshold 0.50	0.5	0.782	0.826	0.966	0.803	0.79	0.218	0.034
Platt-calibrated XGBoost, sensitivity-targeted	0.324	0.848	0.748	0.94	0.795	0.826	0.152	0.06
Platt-calibrated XGBoost, F2-optimized	0.138	0.945	0.634	0.887	0.759	0.861	0.055	0.113
Platt-calibrated XGBoost, VME: ME cost ratio 2:1	0.073	0.976	0.554	0.836	0.707	0.847	0.024	0.164
Platt-calibrated XGBoost, VME: ME cost ratio 5:1	0.041	0.994	0.503	0.795	0.668	0.831	0.006	0.205
Platt-calibrated XGBoost, VME: ME cost ratio 10:1	0.033	0.997	0.482	0.777	0.65	0.822	0.003	0.223
ENN–BLSMOTE–XGBoost, fixed threshold 0.50	0.5	0.873	0.75	0.939	0.807	0.845	0.127	0.061

Precision (PPV, positive predictive value); VME, very major error rate; ME, major error rate; ENN, Edited Nearest Neighbors; BLSMOTE, Borderline Synthetic Minority Oversampling Technique. Platt-calibrated XGBoost refers to XGBoost probabilities recalibrated using Platt scaling. Thresholds for the sensitivity-targeted, F2-optimized, and cost-sensitive operating points were selected using training-fold predictions and subsequently applied to held-out observations. The sensitivity-targeted threshold was selected to target a sensitivity of at least 0.85 in the training data; therefore, the pooled held-out sensitivity could be slightly below 0.85. VME was calculated as FN/(TP+FN), and ME as FP/(FP+TN). The VME: ME ratios indicate the relative weights assigned to the two error types during exploratory cost-sensitive threshold selection.

At the conventional threshold of 0.50, Platt-calibrated XGBoost achieved a sensitivity of 0.782, precision of 0.826, specificity of 0.966, and F2-score of 0.790. ENN–BLSMOTE–XGBoost shifted the operating point toward greater resistant-case detection, increasing sensitivity to 0.873 and reducing VME from 0.218 to 0.127. This gain was accompanied by lower precision (0.750 vs. 0.826), lower specificity (0.939 vs. 0.966), and higher ME (0.061 vs. 0.034), while the F1-score increased slightly from 0.803 to 0.807.

Threshold optimization substantially changed the operating characteristics of the calibrated baseline without modifying the training distribution. The sensitivity-targeted threshold produced sensitivity, precision, specificity, and ME values of 0.848, 0.748, 0.940, and 0.060, respectively, which were close to those of ENN–BLSMOTE–XGBoost at the 0.50 threshold. The resampled model retained moderately higher sensitivity (0.873 vs. 0.848), F1-score (0.807 vs. 0.795), and F2-score (0.845 vs. 0.826), together with a lower VME (0.127 vs. 0.152), whereas precision, specificity, and ME were nearly identical between the two operating points.

More aggressive threshold optimization further reduced missed resistant cases but increased the false-positive burden. F2 optimization increased sensitivity to 0.945 and reduced VME to 0.055, exceeding the corresponding values of ENN–BLSMOTE–XGBoost; however, precision and specificity decreased to 0.634 and 0.887, respectively, and ME increased to 0.113. Similarly, increasing the relative cost assigned to VME from 2:1 to 10:1 progressively increased sensitivity from 0.976 to 0.997 and reduced VME from 0.024 to 0.003, but this was accompanied by decreasing precision, specificity, F1-score, and F2-score and increasing ME.

Overall, these descriptive comparisons indicate that a substantial proportion of the sensitivity gain observed after ENN–BLSMOTE resampling could also be obtained through threshold adjustment of the calibrated baseline. ENN–BLSMOTE–XGBoost at the 0.50 cutoff provided a comparatively balanced sensitivity-oriented operating point, whereas more aggressive threshold optimization could achieve higher sensitivity and lower VME only at the cost of substantially more false-positive resistance predictions. Therefore, the effect of resampling should be interpreted primarily as a change in the sensitivity–false-positive trade-off.

### 3.7 Prediction-anchor and feature-restriction sensitivity analysis

Four nested feature sets were evaluated using the same patient-grouped cross-validation splits and modeling procedures: (A) the complete set of 56 predictors available at or before the prediction anchor; (B) 53 predictors after excluding the durations of carbapenem, β-Lactamase Inhibitor Combinations, and quinolone use; (C) 50 predictors after additionally excluding device-duration variables; and (D) 36 predictors after excluding all duration-based variables. Both XGBoost and ENN–BLSMOTE–XGBoost were refitted separately for each feature set. Performance was assessed using ROC AUC, PR AUC, sensitivity, PPV, specificity, F1-score, F2-score, VME, ME, and Brier score (**Table 5**).

**Table 5:** Feature-restriction sensitivity analysis.

Feature set	Model	No. features	ROC AUC (95% CI)	PR AUC (95% CI)	Sensitivity	PPV	Specificity	F1	F2	VME	ME	Brier
A	XGBoost	56	0.977 (0.974–0.981)	0.912 (0.899–0.925)	0.740	0.869	0.977	0.799	0.762	0.260	0.023	0.045
A	ENN–BLSMOTE–XGBoost	56	0.975 (0.971–0.978)	0.900 (0.886–0.915)	0.848	0.748	0.940	0.795	0.826	0.152	0.060	0.053
B	XGBoost	53	0.967 (0.963–0.971)	0.880 (0.865–0.896)	0.672	0.845	0.974	0.748	0.700	0.328	0.026	0.054
B	ENN–BLSMOTE–XGBoost	53	0.965 (0.961–0.969)	0.870 (0.854–0.887)	0.816	0.704	0.928	0.756	0.791	0.184	0.072	0.064
C	XGBoost	50	0.956 (0.951–0.963)	0.868 (0.850–0.884)	0.676	0.866	0.978	0.759	0.707	0.324	0.022	0.055
C	ENN–BLSMOTE–XGBoost	50	0.951 (0.946–0.958)	0.851 (0.834–0.870)	0.795	0.696	0.928	0.742	0.773	0.205	0.072	0.067
D	XGBoost	36	0.943 (0.936–0.951)	0.839 (0.820–0.858)	0.655	0.843	0.975	0.737	0.685	0.345	0.025	0.062
D	ENN–BLSMOTE–XGBoost	36	0.940 (0.933–0.948)	0.833 (0.814–0.853)	0.765	0.670	0.922	0.714	0.744	0.235	0.078	0.074

Feature set A included all predictors frozen at or before the prediction anchor. Feature set B excluded the durations of carbapenem, β-lactam/β-lactamase-inhibitor combination, and quinolone use. Feature set C additionally excluded device-duration variables. Feature set D excluded all duration-based predictors. Both pipelines were refitted using identical patient-grouped cross-validation splits. ROC AUC and PR AUC are reported with 95% confidence intervals. VME was defined as FN/(TP+FN), and ME as FP/(TN+FP). Threshold-dependent metrics were calculated at 0.50.

Excluding the three treatment-pathway duration variables reduced the ROC AUC of XGBoost from 0.977 to 0.967 and its PR AUC from 0.912 to 0.880. Corresponding values for ENN–BLSMOTE–XGBoost decreased from 0.975 to 0.965 and from 0.900 to 0.870, respectively.

Further exclusion of treatment- and device-duration variables reduced the ROC AUC to 0.956 for XGBoost and 0.951 for ENN–BLSMOTE–XGBoost. When all duration variables were removed, the two pipelines retained ROC AUCs of 0.943 and 0.940 and PR AUCs of 0.839 and 0.833, respectively. Thus, discrimination decreased progressively as duration-based information was removed but remained substantial in the fully duration-restricted analysis.

The operating-point trade-off between the two pipelines was also preserved. With all duration variables excluded, XGBoost maintained higher PPV and specificity and a lower ME, whereas ENN–BLSMOTE–XGBoost maintained higher sensitivity and a lower VME. These findings indicate that the predictive performance was not solely attributable to treatment-pathway or duration features, although these variables contributed meaningful incremental information.

### 3.8 Internal temporal validation

To evaluate temporal transportability, the finalized modeling pipelines were applied to a later same-center cohort collected between January and November 2025. The temporal cohort comprised 1,021 records, including 207 carbapenem-resistant and 814 carbapenem-susceptible records, corresponding to a resistance prevalence of 20.3%. All preprocessing transformations, model parameters, Platt calibration mappings, and operating thresholds had been determined using the historical 2014–2024 cohort and were applied to the temporal cohort without refitting or adjustment. The temporal-validation results are summarized in **Table 6** and visualized in **Figure 5**.

**Table 6:** Discrimination, threshold-dependent classification performance, and probability calibration of the candidate models in the same-center internal temporal validation cohort.

Model	ROC AUC	PR AUC	Recall	Precision	Specificity	Accuracy	F1	F2	VME	ME	Brier score	Calibration intercept	Calibration slope	ECE
Raw XGBoost	0.892 (95% CI 0.866–0.916)	0.705 (95% CI 0.636–0.773)	0.589	0.705	0.937	0.867	0.642	0.609	0.411	0.063	0.106	-0.186	0.400	0.080
Platt-calibrated XGBoost	0.892 (95% CI 0.866–0.916)	0.705 (95% CI 0.636–0.773)	0.705	0.629	0.894	0.856	0.665	0.689	0.295	0.106	0.105	-0.313	0.435	0.076
Raw ENN–BLSMOTE–XGBoost	0.889 (95% CI 0.864–0.912)	0.684 (95% CI 0.613–0.755)	0.715	0.624	0.891	0.855	0.667	0.695	0.285	0.109	0.121	-0.709	0.337	0.108
Platt-calibrated ENN–BLSMOTE–XGBoost	0.889 (95% CI 0.864–0.912)	0.684 (95% CI 0.613–0.755)	0.749	0.605	0.876	0.850	0.670	0.715	0.251	0.124	0.108	-0.382	0.467	0.080

Raw model outputs were evaluated at the default threshold of 0.50. Thresholds for the Platt-calibrated models were selected exclusively from historical out-of-fold predictions to target sensitivity ≥0.85 and were applied unchanged to the untouched January–November 2025 temporal cohort. Platt scaling preserved risk ranking; therefore, calibrated and uncalibrated versions of the same underlying model had identical ROC AUCs and PR AUCs. Ideal calibration corresponds to an intercept of 0 and a slope of 1; lower Brier scores and ECE values indicate better calibration. ROC AUC and PR AUC 95% CIs were estimated using 1,000 patient-cluster bootstrap resamples.

**Figure 5:**
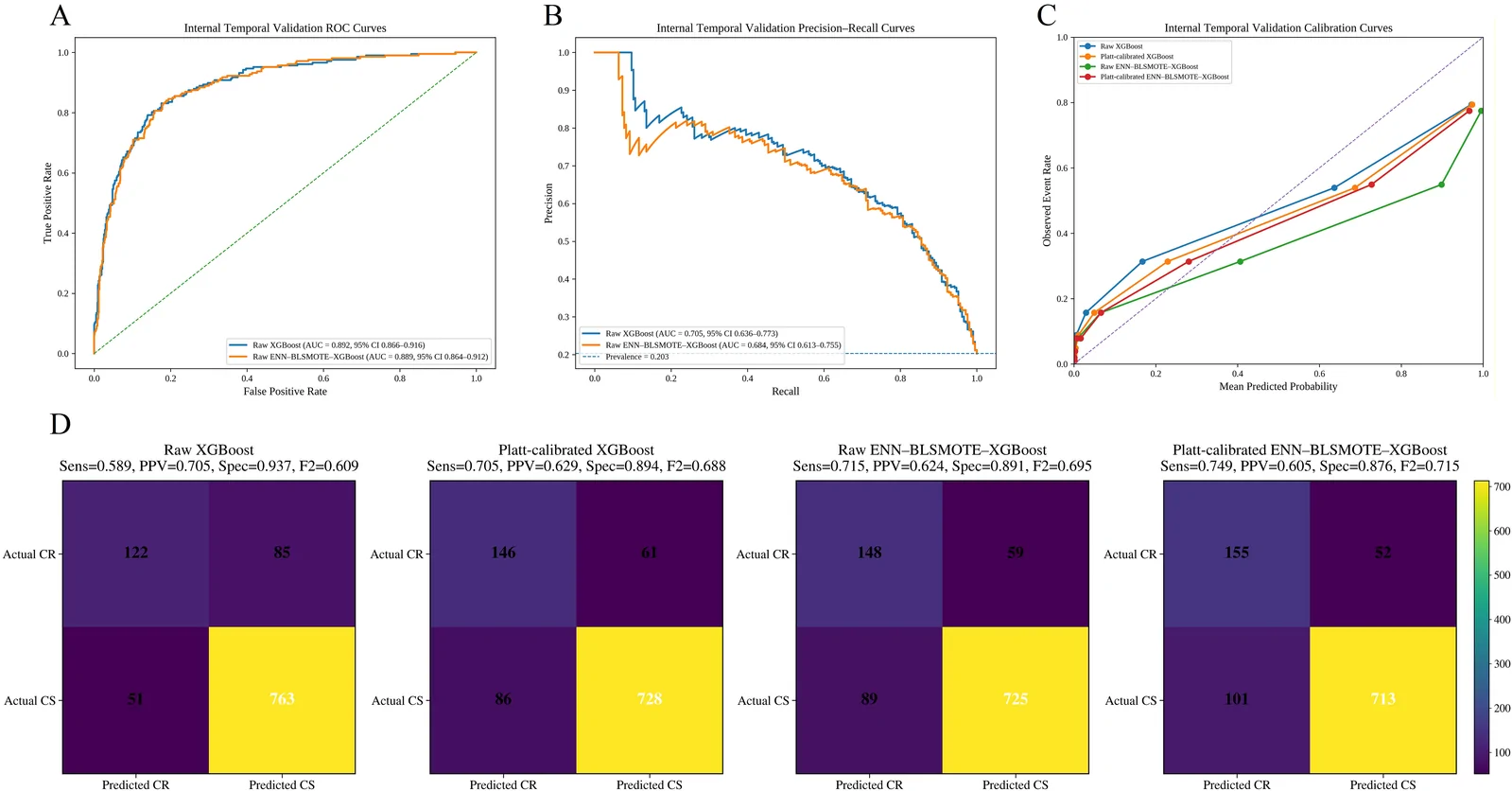
Discrimination, calibration, and classification performance in the same-center internal temporal validation cohort. **(A)** ROC curves for raw XGBoost and raw ENN–BLSMOTE–XGBoost, with ROC AUC values. **(B)** PR curves for the two modeling pipelines, with PR AUC values. **(C)** Calibration curves for the raw and Platt-calibrated probability outputs; the diagonal dashed line represents perfect calibration. **(D)** Confusion matrices for the raw models at the default threshold of 0.50 and the Platt-calibrated models at sensitivity-oriented thresholds selected from historical out-of-fold predictions. Sensitivity, PPV, specificity, and F2-score are shown above each matrix. CR, carbapenem-resistant; CS, carbapenem-susceptible; ROC, receiver operating characteristic; PR, precision–recall; AUC, area under the curve; CI, confidence interval; PPV, positive predictive value; ENN, Edited Nearest Neighbors; BLSMOTE, Borderline Synthetic Minority Oversampling Technique; XGBoost, eXtreme Gradient Boosting.

XGBoost achieved an ROC AUC of 0.892 (95% CI, 0.866–0.916) and a PR AUC of 0.705 (0.636–0.773). Compared with raw XGBoost at the 0.50 threshold, Platt-calibrated XGBoost at the historically selected sensitivity-oriented threshold increased recall from 0.589 to 0.705 and F2-score from 0.609 to 0.689, while reducing VME from 0.411 to 0.295. This improvement was accompanied by lower precision (0.629 vs 0.705), lower specificity (0.894 vs 0.937), and higher ME (0.106 vs 0.063).

ENN–BLSMOTE–XGBoost achieved an ROC AUC of 0.889 (95% CI, 0.864–0.912) and a PR AUC of 0.684 (0.613–0.755). After calibration and threshold adjustment, recall increased from 0.715 to 0.749, F2-score increased from 0.695 to 0.715, and VME decreased from 0.285 to 0.251, whereas precision decreased from 0.624 to 0.605 and ME increased from 0.109 to 0.124. Compared with calibrated XGBoost, calibrated ENN–BLSMOTE–XGBoost therefore provided higher resistant-case detection but generated more false-positive alerts.

Platt scaling preserved risk ranking. It modestly improved XGBoost calibration and produced a larger improvement for ENN–BLSMOTE–XGBoost, reducing its Brier score from 0.121 to 0.108 and ECE from 0.108 to 0.080. Although the negative calibration intercepts and slopes substantially below 1 indicated residual temporal miscalibration, both pipelines retained useful discrimination. In the intended sensitivity-oriented screening setting, ENN–BLSMOTE–XGBoost showed a clinically relevant advantage by achieving higher recall and F2-score and a lower VME, thereby reducing missed resistant cases.

### 3.9 Model interpretability based on SHAP values

Following performance evaluations, the ENN-BLSMOTE-XGBoost model was selected as the final candidate for carbapenem resistance prediction due to its optimal recall-precision balance. SHAP was used to analyze the interpretability of the model (**Figure 6**; **Supplementary Tables S6**-**S10**), with cross-model comparisons of the top 15 SHAP values used to evaluate the impact of sampling strategies on feature importance.

**Figure 6:**
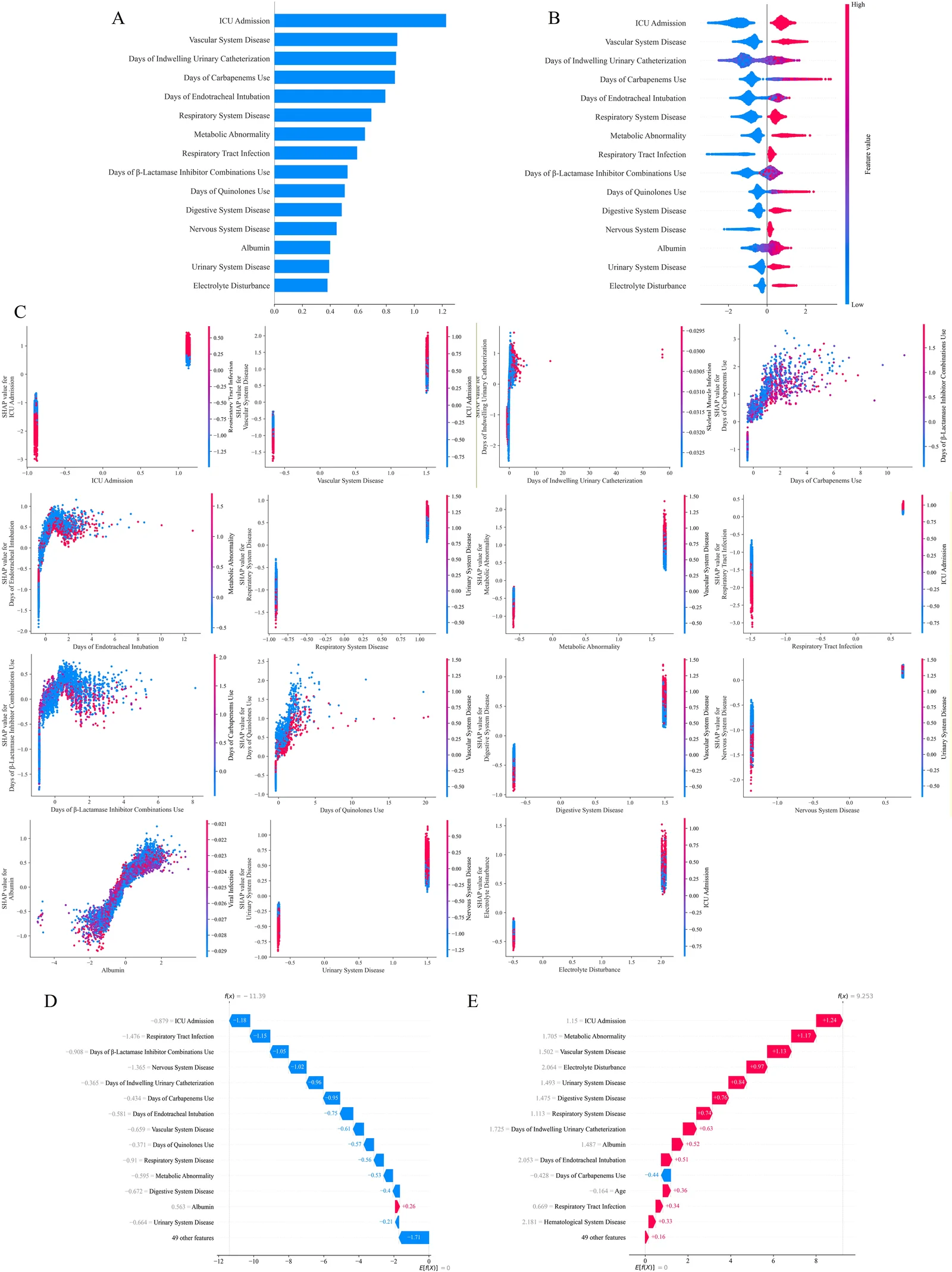
Global and local interpretability of the ENN-BLSMOTE-XGBoost model for carbapenem resistance prediction (Top 15 Features). **(A)** SHAP summary bar plot. Mean absolute SHAP values for the top 15 predictors, reflecting their overall contribution to model output; **(B)** SHAP summary dot plot. Each dot represents a SHAP value for an individual patient. Dot color indicates the actual feature value (red=high, blue=low). Features are ranked by importance; **(C)** SHAP interaction matrix. Pairwise relationships among top features, combining scatter and violin plots to show interaction and distribution; **(D)** SHAP waterfall plot (low-risk individual). Local explanation of feature contributions for a patient predicted to have low risk of carbapenem-resistant *Klebsiella pneumoniae*; **(E)** SHAP waterfall plot (high-risk individual). Local explanation of feature contributions for a patient predicted to have a high risk of carbapenem-resistant *Klebsiella pneumoniae*. ICU, intensive care unit; SHAP, SHapley Additive exPlanations.

As shown in **Figure 6**, ICU admission was the highest-attribution predictor, followed by vascular-system disease and days of indwelling urinary catheterization. Days of carbapenem use and days of endotracheal intubation were also among the highest-attribution features.; The high SHAP attribution of ICU admission likely reflects its role as a composite marker of greater illness severity, invasive procedures, antimicrobial exposure, healthcare-associated infection risk, and other unmeasured dimensions of clinical complexity. Accordingly, its prominence in the model represents a predictive association embedded within the broader ICU care context. The highest-attribution features were broadly consistent across sampling strategies, although modest differences in ranking were observed.

### 3.10 ICU subgroup performance and within-ICU SHAP associations

Because ICU admission was the highest-attribution feature in the full-cohort SHAP analysis, additional analyses were conducted within ICU records to reduce the influence of this subgroup indicator and characterize model performance and predictive associations among ICU patients. The ICU subgroup comprised 2,544 records, including 829 carbapenem-resistant and 1,715 carbapenem-susceptible records, corresponding to a resistance prevalence of 32.6% (**Supplementary Table 11**).

In the ICU-only refit analysis, XGBoost achieved an ROC AUC of 0.956 (95% CI, 0.946–0.965) and a PR AUC of 0.926 (0.902–0.945), whereas ENN–BLSMOTE–XGBoost achieved an ROC AUC of 0.951 (0.940–0.960) and a PR AUC of 0.916 (0.891–0.936) (**Table 7**; **Figures 7A, B**). At the default threshold of 0.50, ENN–BLSMOTE–XGBoost increased sensitivity from 0.762 to 0.872, increased the F2-score from 0.781 to 0.843, and reduced the VME from 0.238 to 0.128. This gain corresponded to 91 additional correctly identified resistant records and 91 fewer false-negative predictions. However, PPV decreased from 0.867 to 0.743, specificity decreased from 0.943 to 0.854, and ME increased from 0.057 to 0.146, indicating a greater false-positive alert burden (**Figure 7C**).

**Table 7:** Discrimination and threshold-dependent performance of XGBoost and ENN–BLSMOTE–XGBoost in the ICU subgroup.

Model	ROC AUC	PR AUC	Recall	PPV	Specificity	F1	F2	VME	ME	Brier score
XGBoost	0.956 (95% CI 0.946–0.965)	0.926 (0.902–0.945)	0.762	0.867	0.943	0.811	0.781	0.238	0.057	0.079
ENN–BLSMOTE–XGBoost	0.951 (95% CI 0.940–0.960)	0.916 (0.891–0.936)	0.872	0.743	0.854	0.802	0.843	0.128	0.146	0.097

ICU, intensive care unit; ROC, receiver operating characteristic; PR, precision–recall; AUC, area under the curve; CI, confidence interval; PPV, positive predictive value; VME, very-major-error rate; ME, major-error rate; ENN, Edited Nearest Neighbors; BLSMOTE, Borderline Synthetic Minority Oversampling Technique; XGBoost, eXtreme Gradient Boosting.

**Figure 7:**
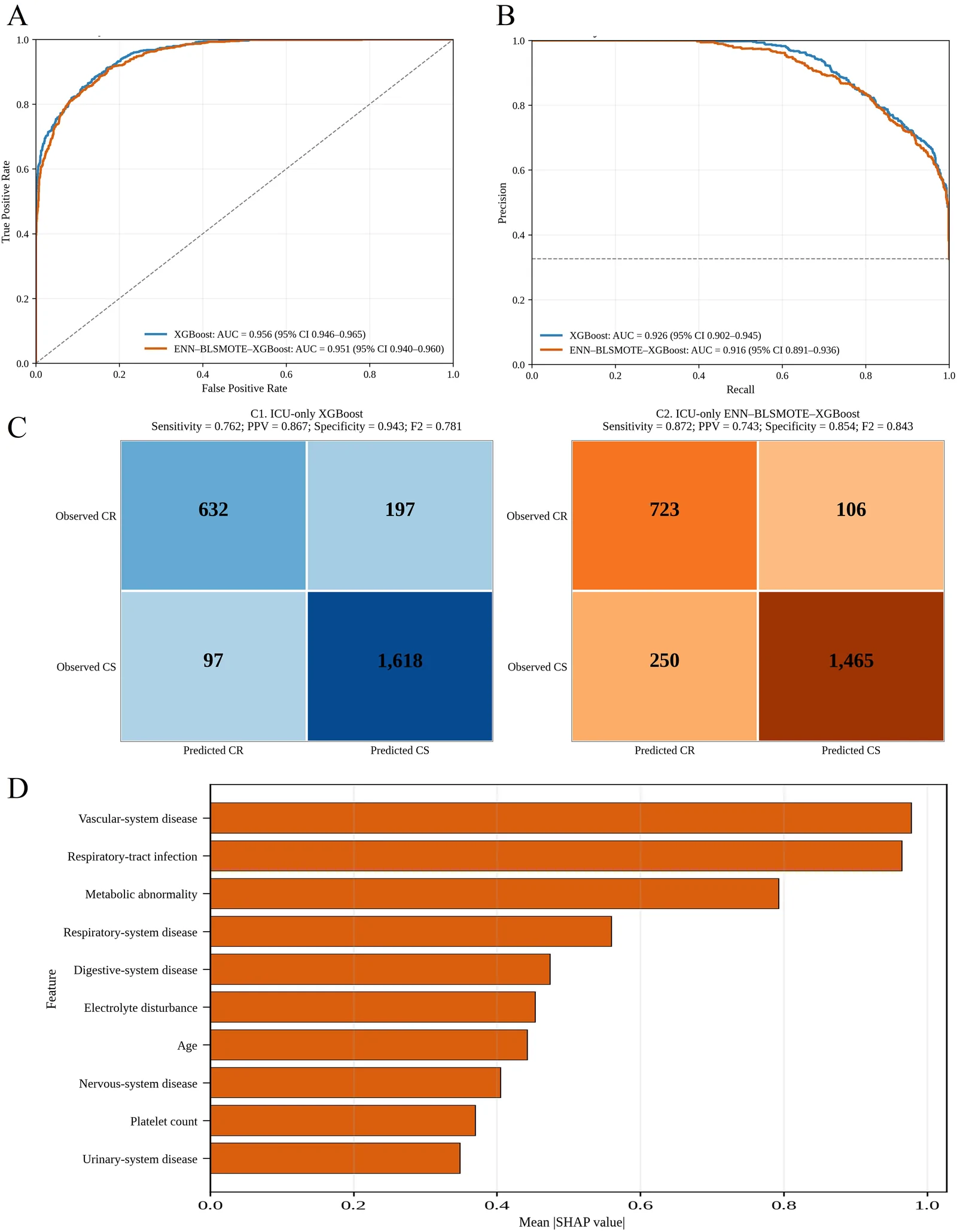
ICU subgroup performance and within-ICU predictive associations. **(A)** SICU-only ROC curves; **(B)** ICU-only PR curves; **(C)** ICU-only confusion matrices; **(D)** ICU-only no-duration SHAP bar plot. Abbreviations: ICU, intensive care unit; ROC, receiver operating characteristic; PR, precision–recall; AUC, area under the curve; CI, confidence interval; BLSMOTE, Borderline Synthetic Minority Oversampling Technique; XGBoost, eXtreme gradient boosting.

Paired subgroup comparisons confirmed the sensitivity-oriented behavior of the resampled model. After multiplicity adjustment, ENN–BLSMOTE–XGBoost retained significantly higher sensitivity and F2 and a lower VME, whereas XGBoost retained higher PPV, specificity, and probability accuracy. The F1-score did not differ significantly between the two models. These findings indicate that ENN–BLSMOTE–XGBoost provided a clinically relevant advantage when minimizing missed resistant ICU cases was prioritized, rather than uniform superiority across all performance dimensions.

In the ICU-only SHAP analysis, ICU admission and all duration variables were excluded. For ENN–BLSMOTE–XGBoost, the highest-attribution features included vascular-system disease, respiratory-tract infection, metabolic abnormality, respiratory-system disease, digestive-system disease, and electrolyte disturbance (**Figure 7D**).

### 3.11 Model performance and probability calibration under varying resistant-to-susceptible patient ratios

Carbapenem resistance prevalence varies regionally due to local antibiotic use, healthcare resources, and demographic differences. Evaluating model robustness across CR: CS ratios is critical for clinical deployment. ENN-BLSMOTE-XGBoost and comparative models were tested across 14 CR: CS ratios (1:1–1:50; **Table 8**; **Supplementary Tables S9**-**S12**; **Supplementary Figures S12**-**S15**).

**Table 8:** Performance metrics of ENN-BLSMOTE-XGBoost under varying Carbapenem-Resistant (CR) vs. Carbapenem-Susceptible (CS) ratios.

CR: CS	Prior ROC AUC	Prior PR AUC	Prior recall	Prior precision	Prior specificity	Prior accuracy	Prior F1	Prior brier	Prior Spiegelhalter Z	Prior Spiegelhalter p
1:1	0.962 (95% CI 0.948–0.965)	0.959 (95% CI 0.944–0.964)	0.840	0.907	0.906	0.879	0.868	0.092	25.724	0.000
1:2	0.969 (95% CI 0.963–0.973)	0.938 (95% CI 0.924–0.947)	0.930	0.794	0.880	0.896	0.856	0.079	29.356	0.000
1:3	0.974 (95% CI 0.971–0.978)	0.927 (95% CI 0.918–0.941)	0.909	0.775	0.912	0.911	0.836	0.064	20.133	0.000
1:4	0.974 (95% CI 0.970–0.978)	0.912 (95% CI 0.898–0.924)	0.870	0.749	0.927	0.916	0.805	0.060	20.619	0.000
1:5	0.977 (95% CI 0.972–0.979)	0.903 (95% CI 0.886–0.914)	0.858	0.738	0.939	0.925	0.790	0.053	18.242	0.000
1:6	0.978 (95% CI 0.975–0.981)	0.890 (95% CI 0.883–0.911)	0.846	0.718	0.946	0.932	0.775	0.046	11.988	0.000
1:7	0.975 (95% CI 0.971–0.978)	0.867 (95% CI 0.856–0.887)	0.815	0.708	0.952	0.935	0.757	0.046	14.263	0.000
1:8	0.976 (95% CI 0.972–0.978)	0.866 (95% CI 0.845–0.878)	0.810	0.686	0.953	0.937	0.742	0.043	11.938	0.000
1:9	0.977 (95% CI 0.973–0.979)	0.862 (95% CI 0.842–0.875)	0.789	0.686	0.960	0.943	0.731	0.039	8.208	0.000
1:10	0.977 (95% CI 0.975–0.980)	0.848 (95% CI 0.835–0.869)	0.793	0.702	0.966	0.951	0.745	0.036	9.046	0.000
1:20	0.979 (95% CI 0.976–0.982)	0.802 (95% CI 0.779–0.820)	0.711	0.688	0.983	0.970	0.696	0.023	-2.802	0.005
1:30	0.978 (95% CI 0.975–0.980)	0.747 (95% CI 0.727–0.773)	0.683	0.628	0.986	0.976	0.654	0.018	-6.949	0.000
1:40	0.978 (95% CI 0.976–0.981)	0.719 (95% CI 0.695–0.744)	0.675	0.566	0.987	0.980	0.615	0.016	-11.039	0.000
1:50	0.976 (95% CI 0.974–0.979)	0.682 (95% CI 0.657–0.710)	0.644	0.517	0.988	0.981	0.573	0.014	-11.184	0.000

As shown in **Table 4** and **Supplementary Figure S7**, ENN-BLSMOTE-XGBoost performed optimally at mildly imbalanced ratios (1:2–1:5), with peak recall=0.930 (1:2) and F-score >0.790, aligning with real-world moderate prevalence scenarios. At severe imbalance (≥1:7), PR AUC decreased from 0.867 to 0.682, recall decreased from 0.815 to 0.644, and specificity increased to 0.988, indicating a shift toward majority-class bias.

Comparative analysis showed ENN-BLSMOTE-XGBoost outperformed other models in mildly imbalanced settings: baseline XGBoost performed best at 1:1 (ROC AUC = 0.972, F1 = 0.910) but deteriorated substantially under extreme imbalance (recall=0.586 at 1:50). SMOTE-TL and BLSMOTE-XGBoost showed superior performance at CR: CS ratios of 1:2–1:5, supporting its potential deployment in moderate-resistance regions, while performance decline at CR: CS ≥1:7 suggests that local model retraining is required for low-prevalence settings.

Spiegelhalter tests were significant at most reconstructed ratios because the same probability mapping was evaluated under substantially different outcome prevalences without ratio-specific updating. This pattern indicates drift in absolute probability calibration rather than a comparable loss of ranking discrimination and was therefore interpreted jointly with the Brier score, ECE, calibration intercept and slope, and calibration curves.

To further assess absolute probability calibration, five representative ratios—1:1, 1:5, 1:10, 1:20, and 1:50—were selected from the 14 scenarios for calibration-transportability analysis (**Supplementary Table 16**). When the nested Platt mapping estimated at the natural development prevalence of 17.24% was applied unchanged, calibration remained well aligned at 1:5, which approximated the development prevalence, with an ECE of 0.0128, calibration intercept of −0.083, and Spiegelhalter *p* = 0.523. In contrast, calibration-in-the-large drift was observed at 1:1, 1:10, 1:20, and 1:50, with intercepts ranging from 1.550 to −2.423 and Spiegelhalter *p* values below 0.05.

Prevalence-prior correction substantially reduced this drift. Across the five representative ratios, corrected calibration intercepts ranged from −0.168 to 0.083, and ECE decreased from 0.1013 to 0.0196 at 1:1, from 0.0308 to 0.0072 at 1:10, from 0.0441 to 0.0062 at 1:20, and from 0.0530 to 0.0031 at 1:50. After prior correction, the Spiegelhalter tests showed no evidence against global calibration in any of the five reconstructed scenarios (all *p*>0.05). Because these cohorts were reconstructed from the same development dataset, these findings support correction of simulated prior-probability shift.

### 3.12 Development of a web-based calculator

To enhance the clinical applicability of the ENN-BLSMOTE-XGBoost model for CRKP prediction, a user-friendly web calculator was developed. Prioritizing predictive performance, computational efficiency, and frontline usability, SFS was used for feature optimization. As shown in **Supplementary Figure S12**, SFS analyzed 15 candidate predictors: cumulative auROC increased sharply initially and plateaued after the 12th feature, leading to the selection of 12 core features to balance accuracy and simplicity.

Accessible at https://crkp-calculator-baxwok2n638p2knfa6fkxb.streamlit.app/, the calculator enables clinicians to input patient-specific values, providing real-time carbapenem resistance probability outputs along with feature contribution visualization to support rapid, evidence-based decision-making.

## 4 Discussion

Machine learning has become a robust tool for antimicrobial resistance prediction (Yurtseven et al., 2023; Babirye et al., 2024; Blechman and Wright, 2024; McMahon et al., 2025), with extensive research optimizing algorithms and feature selection for CRKP prediction. However, few studies have systematically evaluated diverse imbalanced sampling strategies, validated model robustness across resistance prevalence scenarios, or enhanced clinical interpretability, gaps addressed in this study.

Among seven baseline models, XGBoost exhibited the most robust performance (recall=0.730, precision=0.879, ROC AUC = 0.978, PR AUC = 0.916). Its superiority stems from integrated tree pruning, weighted quantile sketch, and gradient boosting (Chen and Guestrin, 2016; Reiner-Benaim et al., 2025), which capture non-linear clinical feature-CRKP relationships while mitigating overfitting, consistent with prior findings (Yang et al., 2016; McGuire et al., 2021; Tran Quoc et al., 2023; Ziaian et al., 2025).

To systematically tackle the class imbalance, we comprehensively evaluated three oversampling, five undersampling, and two hybrid sampling pathways, alongside reversed-order hybrid strategies. Among all approaches, the reversed-order ENN-BLSMOTE hybrid strategy yielded the optimal improvement, boosting XGBoost’s recall to 0.852 while maintaining precision = 0.761 and ROC AUC = 0.976. Existing CRKP prediction studies (Alparslan et al., 2025; Rad et al., 2025) have achieved valuable results but rarely addressed natural data imbalance, whereas our framework ensures high performance under real-world imbalanced distributions. Unlike studies focusing on single strategies (De Waele et al., 2018; Soladoye et al., 2025; Velez et al., 2025), we found that oversampling showed modest gains due to limitations in learning hard-to-classify samples, aggressive undersampling (e.g., NM1) reduced precision unacceptably, and “oversampling-first” hybrid approaches failed to adequately mitigate synthetic noise. In contrast, the ENN-BLSMOTE approach demonstrated synergistic effects through noise reduction and targeted minority class enrichment, highlighting the critical importance of sampling order.

The comparison with Platt-calibrated XGBoost further clarified this finding. By adjusting the threshold without changing the training distribution, the sensitivity-targeted baseline achieved sensitivity, PPV, specificity, and ME values close to those of ENN–BLSMOTE–XGBoost at the 0.50 threshold. More aggressive F2-maximizing or VME-weighted thresholds further reduced missed resistant cases but substantially increased the false-positive burden. Consistent with the approach implemented in ResPredAI (Bonazzetti et al., 2025), these findings indicate that threshold optimization provides a clinically meaningful alternative to resampling. The choice between calibrated XGBoost and ENN–BLSMOTE–XGBoost should consequently depend on the relative clinical consequences of missed resistance and unnecessary resistance alerts.

Although these restrictions reduce temporal leakage, treatment-pathway variables may still reflect confounding by indication. Prior carbapenem,β-Lactamase Inhibitor Combinations, or quinolone exposure may partly encode clinicians’ pre-existing suspicion of resistant infection, whereas device durations may represent accumulated illness severity and healthcare exposure. The feature-restriction analyses therefore provide an important assessment of model dependence on these variables. After all duration-based predictors were excluded, ENN–BLSMOTE–XGBoost retained an ROC AUC of 0.940 and a PR AUC of 0.833. Thus, duration variables contributed incremental information, but the model’s discrimination was not solely attributable to treatment-pathway or device-duration features.

We further validated model robustness across 14 CR: CS ratios (1:1–1:50). ENN-BLSMOTE-XGBoost performed optimally in clinically representative 1:2–1:5 ratios, aligning with real-world resistance burdens, while performance declined at severe imbalance (≥1:7), underscoring the need for local model retraining in low-resistance settings. Notably, BLSMOTE slightly enhanced baseline XGBoost performance even in balanced 1:1 ratios by refining minority class representation and reducing decision boundary ambiguity between susceptible and resistant cases, suggesting that BLSMOTE may improve model performance even under balanced conditions.

The same-center 2025 temporal cohort provided a more stringent evaluation than random cross-validation because the preprocessing transformations, model parameters, calibration mappings, and thresholds were determined from the historical cohort and applied without refitting. Both pipelines retained useful discrimination, although performance was lower than in development. Platt scaling improved the calibration of ENN–BLSMOTE–XGBoost, reducing its Brier score and ECE, but residual temporal miscalibration remained.

SHAP analysis identified ICU admission as the highest-attribution predictor in the full cohort. This result represents a predictive association rather than a causal effect. ICU admission may act as a composite marker of illness severity, invasive procedures, antimicrobial exposure, healthcare-associated infection risk, and other unmeasured aspects of clinical complexity (Ghamari et al., 2025; Goldschmidt et al., 2025; Liu et al., 2025; Micek et al., 2025). Similarly, the associations of vascular-system disease, catheter duration, and antimicrobial exposure should not be interpreted as independently modifiable causal determinants. In the ICU-only analysis, useful discrimination and predictive associations remained after ICU admission and duration variables were excluded, suggesting that the model captured additional clinical information within this high-risk subgroup.

This study has limitations: single-center retrospective data may restrict generalizability, and model calibration declined at extreme imbalance (≥1:30) without targeted optimization. Future research should incorporate multi-center data for external validation and advanced calibration techniques to improve performance in extreme imbalance scenarios.

## 5 Conclusion

In conclusion, this study systematically addressed class imbalance in CRKP prediction by evaluating seven baseline machine learning models and eighteen sampling strategies, including three oversampling, five undersampling, and ten hybrid strategies, and by validating performance across 14 CR: CS ratios. XGBoost emerged as the most robust baseline model, achieving balanced recall and precision across clinically representative CR: CS ratios via the reversed-order ENN-BLSMOTE hybrid strategy. SHAP analysis identified ICU admission, vascular system disease, days of indwelling urinary catheterization, days of carbapenem use, and days of endotracheal intubation as the top predictors.

This work establishes a comprehensive framework for imbalanced medical data analysis in antimicrobial resistance prediction, improving the identification of patients likely to harbor carbapenem-resistant *K. pneumoniae* isolates and supporting infection prevention, antimicrobial stewardship, and individualized clinical decision-making.

## Data Availability

The data analyzed in this study is subject to the following licenses/restrictions: The datasets are not publicly available due to patient privacy and institutional restrictions. Requests to access these datasets should be directed to Lili Geng, zxyygll@163.com.
