# Supplementary material for: An explainable machine learning approach to predicting carbapenem resistance in Klebsiella pneumoniae with imbalanced data and resistance-ratio validation
Source: Front Cell Infect Microbiol. 2026 Sep 16;16:1906306. doi: 10.3389/fcimb.2026.1906306 (PMC13623693; doi:10.3389/fcimb.2026.1906306)
Supplement: Supplementary file 1 [file Supplementaryfile1.docx]

Supplementary Material

**Supplementary Table S1.** Patient Characteristic Information.

**Supplementary Table S2.** Baseline model hyperparameter settings.

**Supplementary Table S3.** Performance of Hybrid Sampling-Optimized Models (5-Fold Cross-Validation).

**Supplementary Fig. S1. Performance Visualization of Hybrid Sampling Pathway A-Optimized Models.**

**Supplementary Fig. S2. Performance Visualization of Hybrid Sampling Pathway B-Optimized Models.**

**Supplementary Table S4.** Performance of GBDT-Based Sampling Extensions.

**Supplementary Table S5.** Multiplicity-Adjusted Key Model Comparisons.

**Supplementary Table S6.** Top 15 Feature Mean SHAP Values of ENN-BLSMOTE-XGBoost.

**Supplementary Table S7.** Top 15 Feature Mean SHAP Values of SMOTE-TL-XGBoost.

**Supplementary Table S8.** Top 15 Feature Mean SHAP Values of ENN-XGBoost.

**Supplementary Table S9.** Top 15 Feature Mean SHAP Values of BLSMOTE-XGBoost.

**Supplementary Table S10.** Top 15 Feature Mean SHAP Values of XGBoost.

**Supplementary Table S11.** ICU and Non-ICU Cohort Compositions.

**Supplementary Fig. S3. SHAP Summary Dot Plot of SMOTE-TL-XGBoost (Top 15 Features).**

**Supplementary Fig. S4. SHAP Summary Dot Plot of ENN-XGBoost (Top 15 Features).**

**Supplementary Fig. S5. SHAP Summary Dot Plot of BLSMOTE-XGBoost (Top 15 Features).**

**Supplementary Fig. S6. SHAP Summary Dot Plot of XGBoost (Top 15 Features).**

**Supplementary Table S12** Performance **Metrics** of SMOTE-TL-XGBoost for Klebsiella pneumoniae Carbapenem Resistance Prediction Under Varying CR:CS Ratios.

**Supplementary Table S13.** Performance Metrics of ENN-XGBoost for Klebsiella pneumoniae Carbapenem Resistance Prediction Under Varying CR:CS Ratios.

**Supplementary Table S14.** Performance Metrics of BLSMOTE-XGBoost for Klebsiella pneumoniae Carbapenem Resistance Prediction Under Varying CR:CS Ratios.

**Supplementary Table S16.** Performance Metrics of Baseline XGBoost for Klebsiella pneumoniae Carbapenem Resistance Prediction Under Varying CR:CS Ratios.

**Supplementary Table S16.** Calibration Transportability and Prevalence-Prior Correction Across Five Representative CR:CS Ratios.

**Supplementary Fig. S7.** Performance of ENN-BLSMOTE-XGBoost Under Varying CR:CS Ratios.

**Supplementary Fig. S8. Performance of XGBoost Under Varying CR:CS Ratios.**

**Supplementary Fig. S9. Performance of SMOTE-TL-XGBoost Under Varying CR:CS Ratios.**

**Supplementary Fig. S10. Performance of ENN-XGBoost Under Varying CR:CS Ratios.**

**Supplementary Fig. S11. Performance of BLSMOTE-XGBoost Under Varying CR:CS Ratios.**

**Supplementary Fig. S12.** Sequential Forward Selection (SFS) for Feature Optimization in the ENN-BLSMOTE-XGBoost Model.

# Supplementary Table S1. Patient Characteristic Information.

| **Variable** | **CR cases** | **CS cases** | **P** |
| --- | --- | --- | --- |
|  | n=999 | n=4795 |  |
| Gender (Female) ^a^ | 324 (32.4) | 1462 (30.5) | 0.241 |
| Age ^b^ | 82.00 (72.00-91.00) | 76.00 (66.00-88.00) | <0.001 |
| ICU Admission ^a^ | 829 (83.0) | 1715 (35.8) | <0.001 |
| Lymphocyte (×10⁹/L) ^b^ | 1.30 (0.91-1.80) | 1.21 (0.84-1.70) | <0.001 |
| Albumin (g/L) ^b^ | 32.80 (28.90-37.00) | 33.50 (29.10-38.00) | 0.012 |
| Platelet (×10⁹/L) ^b^ | 216.50 (159.00-275.75) | 204.00 (148.00-269.00) | 0.001 |
| Days of Aminoglycosides Use ^c^ | 4.90 (3.87-9.52) (95.3%) | 3.87 (2.11-7.39) (97.7%) | <0.001 |
| Days of Macrolides Use ^c^ | 3.99 (2.93-6.47) (97.8%) | 2.99 (2.02-3.91) (98.5%) | 0.093 |
| Days of β-Lactamase Inhibitor Combinations Use ^b^ | 4.95 (2.49-7.69) | 2.01 (0.00-4.81) | <0.001 |
| Days of Antifungal Agents Use ^c^ | 4.98 (2.92-8.08) (76.5%) | 3.89 (1.91-6.97) (92.6%) | <0.001 |
| Days of Quinolones Use ^c^ | 5.00 (2.66-7.88) (57.9%) | 3.55 (1.98-5.11) (77.7%) | <0.001 |
| Days of Lincosamides Use ^c^ | 4.20 (4.20-4.20) (99.9%) | 3.85 (1.78-6.07) (99.7%) | 0.317 |
| Days of Penicillins Use ^c^ | 5.92 (2.75-8.77) (85.2%) | 3.81 (2.13-5.17) (82.5%) | 0.133 |
| Days of Tetracyclines Use ^c^ | 3.38 (1.88-9.00) (98.0%) | 2.56 (2.28-2.84) (100.0%) | <0.001 |
| Days of Carbapenems Use ^b^ | 1.98 (0.00-5.90) | 0.00 (0.00-0.00) | <0.001 |
| Days of Glycopeptides Use ^c^ | 4.03 (2.01-7.00) (82.3%) | 3.42 (1.91-6.79) (94.3%) | <0.001 |
| Days of First-Generation Cephalosporins Use ^c^ | 3.78 (1.76-4.60) (92.5%) | 2.89 (1.69-4.92) (92.0%) | 0.646 |
| Days of Second-Generation Cephalosporins Use ^c^ | 6.01 (1.81-11.78) (94.5%) | 2.97 (1.91-4.88) (94.7%) | 0.720 |
| Days of Third-Generation Cephalosporins Use ^c^ | 5.76 (2.74-9.06) (81.4%) | 3.44 (1.92-5.68) (89.5%) | <0.001 |
| Days of Fourth-Generation Cephalosporins Use ^c^ | 2.75 (1.96-8.83) (99.7%) | 4.93 (2.98-6.81) (99.6%) | 0.592 |
| Days of Cephamycins Use ^c^ | 6.89 (2.82-12.78) (98.0%) | 3.69 (2.38-4.95) (95.6%) | <0.001 |
| Days of Nitroimidazoles Use ^c^ | 5.38 (2.16-11.72) (96.5%) | 3.59 (2.00-5.75) (95.1%) | 0.056 |
| Metabolic Abnormality ^a^ | 607 (60.8) | 888 (18.5) | <0.001 |
| Electrolyte Disturbance ^a^ | 461 (46.1) | 655 (13.7) | <0.001 |
| Malignant Tumor ^a^ | 143 (14.3) | 470 (9.8) | <0.001 |
| Circulatory System Disease ^a^ | 899 (90.0) | 3195 (66.6) | <0.001 |
| Respiratory System Disease ^a^ | 788 (78.9) | 1828 (38.1) | <0.001 |
| Urinary System Disease ^a^ | 615 (61.6) | 1170 (24.4) | <0.001 |
| Endocrine System Disease ^a^ | 146 (14.6) | 192 (4.0) | <0.001 |
| Nervous System Disease ^a^ | 913 (91.4) | 2836 (59.1) | <0.001 |
| Diabetes Mellitus ^a^ | 403 (40.3) | 1333 (27.8) | <0.001 |
| Trauma ^a^ | 162 (16.2) | 358 (7.5) | <0.001 |
| Digestive System Disease ^a^ | 649 (65.0) | 1147 (23.9) | <0.001 |
| Hematological System Disease ^a^ | 418 (41.8) | 595 (12.4) | <0.001 |
| Vascular System Disease ^a^ | 659 (66.0) | 1101 (23.0) | <0.001 |
| Multiple Organ Dysfunction Syndrome (MODS) ^a^ | 17 (1.7) | 25 (0.5) | <0.001 |
| Rheumatic Immune System Disease ^a^ | 37 (3.7) | 86 (1.8) | <0.001 |
| Otorhinolaryngological Disease ^a^ | 4 (0.4) | 7 (0.1) | 0.105 |
| Ophthalmic Disease ^a^ | 10 (1.0) | 7 (0.1) | <0.001 |
| Biliary Tract Infection ^a^ | 133 (13.3) | 166 (3.5) | <0.001 |
| Infective Endocarditis ^a^ | 1 (0.1) | 1 (0.0) | 0.315 |
| Respiratory Tract Infection ^a^ | 955 (95.6) | 3030 (63.2) | <0.001 |
| Intracranial Infection ^a^ | 23 (2.3) | 43 (0.9) | <0.001 |
| Urinary Tract Infection ^a^ | 321 (32.1) | 359 (7.5) | <0.001 |
| Skin Infection ^a^ | 110 (11.0) | 138 (2.9) | <0.001 |
| Digestive Tract Infection ^a^ | 223 (22.3) | 411 (8.6) | <0.001 |
| Bloodstream Infection ^a^ | 57 (5.7) | 80 (1.7) | <0.001 |
| Surgical Site Infection ^a^ | 3 (0.3) | 19 (0.4) | 1.000 |
| Ocular Infection ^a^ | 1 (0.1) | 4 (0.1) | 1.000 |
| Skeletal Muscle Infection ^a^ | 2 (0.2) | 4 (0.1) | 0.277 |
| Surgery ^a^ | 161 (16.1) | 138 (2.9) | <0.001 |
| Implantation ^a^ | 71 (7.1) | 99 (2.1) | <0.001 |
| Chemotherapy ^a^ | 11 (1.1) | 56 (1.2) | 0.986 |
| Days of Indwelling Urinary Catheterization ^b^ | 9.85 (5.74-16.54) | 2.71 (0.00-5.89) | <0.001 |
| Days of Drainage ^c^ | 4.62 (1.91-7.34) (85.9%) | 3.34 (1.98-4.74) (91.2%) | <0.001 |
| Days of Endotracheal Intubation ^b^ | 4.09 (0.00-7.06) | 0.00 (0.00-2.30) | <0.001 |

^a^ Discrete variables are presented as number (percentage), i.e., n (%), and P-values were calculated using the Chi-square test. ^b^ Continuous variables are presented as median (Interquartile Range), i.e., median (IQR), and P-values were calculated using the Mann-Whitney U test. ^c^ For variables with a median and IQR of 0 in the full sample, values are presented as the median and IQR among non-zero observations, with the proportion of zero values shown in parentheses. P-values were calculated using the Mann-Whitney U test with consideration of zero values.

# Supplementary Table S2. Baseline model hyperparameter settings.

| Model | Hyperparameter Settings |
| --- | --- |
| XGBoost | n_estimators=300; learning_rate=0.05; max_depth=5; subsample=0.8; colsample_bytree=0.8; random_state=256; n_jobs=-1 |
| LightGBM | n_estimators=300; learning_rate=0.05; max_depth=-1 (no limit); subsample=0.8; colsample_bytree=0.8; random_state=256; n_jobs=-1 |
| RF | n_estimators=300; max_depth=10; random_state=256; n_jobs=-1 |
| LR | max_iter=2000; solver="liblinear" |
| SVM | kernel="rbf"; probability=True; random_state=256 |
| KNN | n_neighbors=5 |
| GBDT | n_estimators=300; learning_rate=0.05; max_depth=5; random_state =256 |

XGBoost, eXtreme Gradient Boosting; LightGBM, Light Gradient Boosting Machine; RF, Random Forest; LR,Logistic Regression; SVM, Support Vector Machine; KNN, K-Nearest Neighbors; GBDT, Gradient Boosting Decision Tree.

# Supplementary Table S3. Performance of Hybrid Sampling-Optimized Models (5-Fold Cross-Validation).

| Model | ROC AUC | PR AUC | Recall | Precision | Specificity | Accuracy | F1 | Brier Score | Spiegelhalter Z | Spiegelhalter p |
| --- | --- | --- | --- | --- | --- | --- | --- | --- | --- | --- |
| SMOTE-TL-XGBoost | 0.979 (95% CI 0.975–0.981) | 0.917 (95% CI 0.903–0.927) | 0.774 | 0.845 | 0.970 | 0.936 | 0.808 | 0.044 | 2.420 | 0.265 |
| SMOTE-TL-LightGBM | 0.978 (95% CI 0.975–0.981) | 0.917 (95% CI 0.904–0.927) | 0.759 | 0.863 | 0.975 | 0.938 | 0.807 | 0.045 | 8.251 | 0.000 |
| SMOTE-TL-RF | 0.971 (95% CI 0.967–0.975) | 0.885 (95% CI 0.870–0.899) | 0.740 | 0.800 | 0.961 | 0.923 | 0.768 | 0.058 | -15.014 | 0.000 |
| SMOTE-ENN-XGBoost | 0.977 (95% CI 0.974–0.980) | 0.912 (95% CI 0.897–0.922) | 0.743 | 0.861 | 0.975 | 0.935 | 0.797 | 0.046 | 4.233 | 0.056 |
| SMOTE-ENN-LightGBM | 0.977 (95% CI 0.973–0.979) | 0.912 (95% CI 0.898–0.922) | 0.736 | 0.864 | 0.976 | 0.934 | 0.794 | 0.048 | 11.100 | 0.000 |
| SMOTE-ENN-RF | 0.970 (95% CI 0.966–0.974) | 0.884 (95% CI 0.868–0.897) | 0.707 | 0.803 | 0.964 | 0.919 | 0.751 | 0.057 | -14.166 | 0.000 |
| BLSMOTE-ENN-XGBoost | 0.978 (95% CI 0.974–0.981) | 0.914 (95% CI 0.900–0.924) | 0.753 | 0.862 | 0.975 | 0.936 | 0.803 | 0.045 | 3.892 | 0.005 |
| BLSMOTE-ENN-LightGBM | 0.977 (95% CI 0.974–0.980) | 0.913 (95% CI 0.899–0.924) | 0.741 | 0.871 | 0.977 | 0.936 | 0.801 | 0.047 | 10.936 | 0.000 |
| BLSMOTE-ENN-RF | 0.968 (95% CI 0.964–0.972) | 0.875 (95% CI 0.859–0.890) | 0.732 | 0.793 | 0.960 | 0.921 | 0.761 | 0.059 | -13.964 | 0.000 |
| BLSMOTE-TL-XGBoost | 0.978 (95% CI 0.975–0.981) | 0.915 (95% CI 0.901–0.925) | 0.770 | 0.841 | 0.969 | 0.935 | 0.803 | 0.045 | 2.747 | 0.163 |
| BLSMOTE-TL-LightGBM | 0.978 (95% CI 0.975–0.981) | 0.916 (95% CI 0.903–0.926) | 0.764 | 0.865 | 0.975 | 0.939 | 0.811 | 0.045 | 8.405 | 0.000 |
| BLSMOTE-TL-RF | 0.968 (95% CI 0.964–0.972) | 0.871 (95% CI 0.855–0.887) | 0.751 | 0.773 | 0.954 | 0.919 | 0.762 | 0.060 | -14.239 | 0.000 |
| ADASYN-TL-XGBoost | 0.979 (95% CI 0.975–0.981) | 0.917 (95% CI 0.903–0.927) | 0.771 | 0.844 | 0.970 | 0.936 | 0.805 | 0.044 | 2.197 | 0.351 |
| ADASYN-TL-LightGBM | 0.978 (95% CI 0.974–0.981) | 0.915 (95% CI 0.902–0.926) | 0.765 | 0.859 | 0.974 | 0.938 | 0.809 | 0.046 | 8.176 | 0.000 |
| ADASYN-TL-RF | 0.968 (95% CI 0.964–0.972) | 0.872 (95% CI 0.856–0.888) | 0.775 | 0.760 | 0.949 | 0.919 | 0.767 | 0.061 | -14.515 | 0.000 |
| SMOTE-NCL-XGBoost | 0.978 (95% CI 0.975–0.981) | 0.916 (95% CI 0.902–0.927) | 0.749 | 0.861 | 0.975 | 0.936 | 0.801 | 0.045 | 4.423 | 0.010 |
| SMOTE-NCL-LightGBM | 0.978 (95% CI 0.974–0.981) | 0.916 (95% CI 0.903–0.927) | 0.730 | 0.882 | 0.980 | 0.936 | 0.798 | 0.047 | 11.009 | 0.000 |
| SMOTE-NCL-RF | 0.972 (95% CI 0.968–0.975) | 0.891 (95% CI 0.876–0.904) | 0.670 | 0.846 | 0.975 | 0.922 | 0.747 | 0.056 | -13.993 | 0.000 |
| SMOTE-NM1-XGBoost | 0.979 (95% CI 0.975–0.981) | 0.916 (95% CI 0.902–0.927) | 0.773 | 0.837 | 0.969 | 0.935 | 0.803 | 0.044 | 2.445 | 0.313 |
| SMOTE-NM1-LightGBM | 0.979 (95% CI 0.975–0.981) | 0.919 (95% CI 0.905–0.928) | 0.759 | 0.859 | 0.974 | 0.937 | 0.806 | 0.045 | 8.160 | 0.000 |
| SMOTE-NM1-RF | 0.970 (95% CI 0.966–0.974) | 0.883 (95% CI 0.868–0.897) | 0.733 | 0.795 | 0.960 | 0.921 | 0.762 | 0.058 | -14.953 | 0.000 |
| ADASYN-NM1-XGBoost | 0.978 (95% CI 0.974–0.981) | 0.914 (95% CI 0.900–0.924) | 0.773 | 0.838 | 0.969 | 0.935 | 0.804 | 0.045 | 2.511 | 0.343 |
| ADASYN-NM1-LightGBM | 0.979 (95% CI 0.975–0.981) | 0.918 (95% CI 0.904–0.928) | 0.765 | 0.866 | 0.975 | 0.939 | 0.812 | 0.045 | 7.893 | 0.000 |
| ADASYN-NM1-RF | 0.967 (95% CI 0.963–0.971) | 0.869 (95% CI 0.853–0.885) | 0.775 | 0.757 | 0.948 | 0.918 | 0.766 | 0.062 | -14.249 | 0.000 |
| ENN-BLSMOTE-XGBoost | 0.976 (95% CI 0.972–0.979) | 0.906 (95% CI 0.891–0.917) | 0.852 | 0.761 | 0.944 | 0.928 | 0.804 | 0.051 | 6.146 | 0.001 |
| ENN-BLSMOTE-LightGBM | 0.976 (95% CI 0.972–0.979) | 0.905 (95% CI 0.889–0.915) | 0.839 | 0.770 | 0.947 | 0.929 | 0.802 | 0.053 | 14.032 | 0.000 |
| ENN-BLSMOTE-RF | 0.966 (95% CI 0.962–0.970) | 0.864 (95% CI 0.847–0.880) | 0.830 | 0.712 | 0.930 | 0.913 | 0.766 | 0.065 | -13.267 | 0.000 |
| NM1-SMOTE-XGBoost | 0.956 (95% CI 0.949–0.961) | 0.850 (95% CI 0.826–0.864) | 0.922 | 0.533 | 0.832 | 0.847 | 0.675 | 0.114 | 23.913 | 0.000 |
| NM1-SMOTE-LightGBM | 0.950 (95% CI 0.943–0.956) | 0.833 (95% CI 0.810–0.850) | 0.923 | 0.507 | 0.812 | 0.832 | 0.654 | 0.137 | 48.185 | 0.000 |
| NM1-SMOTE-RF | 0.933 (95% CI 0.924–0.941) | 0.808 (95% CI 0.783–0.827) | 0.899 | 0.464 | 0.783 | 0.803 | 0.612 | 0.132 | -8.470 | 0.000 |

SMOTE, Synthetic Minority Oversampling Technique; BLSMOTE, Borderline Synthetic Minority Oversampling Technique; ADASYN, Adaptive Synthetic Sampling; TL, Tomek Links; ENN, Edited Nearest Neighbors; NCL, Neighborhood Cleaning Rule; NM1, Neighborhood Majority 1; XGBoost, eXtreme Gradient Boosting; LightGBM, Light Gradient Boosting Machine; RF, Random Forest.

# Supplementary Table S4. Performance of GBDT-Based Sampling Extensions.

| Model | ROC AUC (95% CI) | PR AUC (95% CI) | Recall | Precision | Specificity | Accuracy | F1 | F2 | VME | ME | Brier Score |
| --- | --- | --- | --- | --- | --- | --- | --- | --- | --- | --- | --- |
| BLSMOTE-TL-GBDT | 0.975 (95% CI 0.972–0.979) | 0.903 (95% CI 0.889–0.917) | 0.765 | 0.814 | 0.964 | 0.929 | 0.788 | 0.774 | 0.235 | 0.036 | 0.048 |
| SMOTE-GBDT | 0.975 (95% CI 0.971–0.978) | 0.902 (95% CI 0.888–0.916) | 0.765 | 0.820 | 0.965 | 0.930 | 0.791 | 0.775 | 0.235 | 0.035 | 0.049 |
| BLSMOTE-GBDT | 0.975 (95% CI 0.971–0.978) | 0.902 (95% CI 0.887–0.916) | 0.759 | 0.811 | 0.963 | 0.928 | 0.784 | 0.769 | 0.241 | 0.037 | 0.048 |
| SMOTE-TL-GBDT | 0.974 (95% CI 0.971–0.978) | 0.901 (95% CI 0.886–0.915) | 0.762 | 0.812 | 0.963 | 0.929 | 0.786 | 0.771 | 0.238 | 0.037 | 0.049 |
| SMOTE-NCL-GBDT | 0.975 (95% CI 0.971–0.978) | 0.901 (95% CI 0.887–0.914) | 0.732 | 0.849 | 0.973 | 0.931 | 0.786 | 0.753 | 0.268 | 0.027 | 0.049 |
| SMOTE-NM1-GBDT | 0.974 (95% CI 0.971–0.978) | 0.901 (95% CI 0.886–0.914) | 0.762 | 0.817 | 0.964 | 0.929 | 0.788 | 0.772 | 0.238 | 0.036 | 0.049 |
| ADASYN-GBDT | 0.975 (95% CI 0.971–0.978) | 0.901 (95% CI 0.885–0.915) | 0.775 | 0.809 | 0.962 | 0.930 | 0.791 | 0.781 | 0.225 | 0.038 | 0.049 |
| ADASYN-NM1-GBDT | 0.974 (95% CI 0.971–0.978) | 0.900 (95% CI 0.885–0.915) | 0.770 | 0.821 | 0.965 | 0.931 | 0.794 | 0.779 | 0.230 | 0.035 | 0.049 |
| ADASYN-TL-GBDT | 0.974 (95% CI 0.971–0.978) | 0.899 (95% CI 0.883–0.914) | 0.774 | 0.810 | 0.962 | 0.930 | 0.792 | 0.781 | 0.226 | 0.038 | 0.049 |
| BLSMOTE-ENN-GBDT | 0.974 (95% CI 0.970–0.977) | 0.899 (95% CI 0.884–0.913) | 0.738 | 0.823 | 0.967 | 0.927 | 0.778 | 0.753 | 0.262 | 0.033 | 0.050 |
| SMOTE-ENN-GBDT | 0.974 (95% CI 0.970–0.977) | 0.898 (95% CI 0.883–0.912) | 0.753 | 0.823 | 0.966 | 0.929 | 0.786 | 0.766 | 0.247 | 0.034 | 0.049 |
| TL-GBDT | 0.972 (95% CI 0.968–0.976) | 0.892 (95% CI 0.878–0.906) | 0.714 | 0.843 | 0.972 | 0.928 | 0.773 | 0.736 | 0.286 | 0.028 | 0.052 |
| GBDT | 0.972 (95% CI 0.968–0.975) | 0.892 (95% CI 0.877–0.907) | 0.698 | 0.852 | 0.975 | 0.927 | 0.767 | 0.724 | 0.302 | 0.025 | 0.052 |
| OSS-GBDT | 0.971 (95% CI 0.967–0.975) | 0.890 (95% CI 0.875–0.904) | 0.707 | 0.843 | 0.973 | 0.927 | 0.769 | 0.730 | 0.293 | 0.027 | 0.053 |
| ENN-BLSMOTE-GBDT | 0.971 (95% CI 0.967–0.975) | 0.887 (95% CI 0.871–0.903) | 0.838 | 0.732 | 0.936 | 0.919 | 0.782 | 0.814 | 0.162 | 0.064 | 0.059 |
| ENN-GBDT | 0.970 (95% CI 0.966–0.974) | 0.885 (95% CI 0.869–0.900) | 0.794 | 0.775 | 0.952 | 0.925 | 0.784 | 0.790 | 0.206 | 0.048 | 0.056 |
| NCL-GBDT | 0.970 (95% CI 0.966–0.974) | 0.885 (95% CI 0.869–0.900) | 0.758 | 0.791 | 0.958 | 0.924 | 0.774 | 0.764 | 0.242 | 0.042 | 0.054 |
| NM1-GBDT | 0.948 (95% CI 0.941–0.954) | 0.815 (95% CI 0.794–0.837) | 0.914 | 0.517 | 0.822 | 0.838 | 0.660 | 0.792 | 0.086 | 0.178 | 0.125 |
| NM1-SMOTE-GBDT | 0.948 (95% CI 0.941–0.954) | 0.815 (95% CI 0.794–0.837) | 0.914 | 0.517 | 0.822 | 0.838 | 0.660 | 0.792 | 0.086 | 0.178 | 0.125 |

SMOTE, Synthetic Minority Oversampling Technique; BLSMOTE, Borderline Synthetic Minority Oversampling Technique; ADASYN, Adaptive Synthetic Sampling; TL, Tomek Links; ENN, Edited Nearest Neighbors; NCL, Neighborhood Cleaning Rule; NM1, Neighborhood Majority 1; GBDT, Gradient Boosting Decision Tree.

# Supplementary Table S5. Multiplicity-Adjusted Key Model Comparisons.

| Comparison family | Candidate | Reference | Metric | Candidate value | Reference value | Difference | 95% CI | Bootstrap p | BH-FDR p | Bonferroni p | BH-FDR significant | Bonferroni significant |
| --- | --- | --- | --- | --- | --- | --- | --- | --- | --- | --- | --- | --- |
| baseline_vs_XGBoost | LightGBM | XGBoost | ROC AUC | 0.9770 | 0.9774 | -0.0004 | -0.0016 to 0.0009 | 0.5275 | 0.5275 | 1.0000 | FALSE | FALSE |
| baseline_vs_XGBoost | RF | XGBoost | ROC AUC | 0.9691 | 0.9774 | -0.0083 | -0.0116 to -0.0060 | 0.0020 | 0.0024 | 0.0120 | TRUE | TRUE |
| baseline_vs_XGBoost | LR | XGBoost | ROC AUC | 0.9653 | 0.9774 | -0.0122 | -0.0170 to -0.0077 | 0.0020 | 0.0024 | 0.0120 | TRUE | TRUE |
| baseline_vs_XGBoost | SVM | XGBoost | ROC AUC | 0.9606 | 0.9774 | -0.0169 | -0.0229 to -0.0122 | 0.0020 | 0.0024 | 0.0120 | TRUE | TRUE |
| baseline_vs_XGBoost | KNN | XGBoost | ROC AUC | 0.8686 | 0.9774 | -0.1089 | -0.1272 to -0.0912 | 0.0020 | 0.0024 | 0.0120 | TRUE | TRUE |
| baseline_vs_XGBoost | GBDT | XGBoost | ROC AUC | 0.9717 | 0.9774 | -0.0057 | -0.0086 to -0.0032 | 0.0020 | 0.0024 | 0.0120 | TRUE | TRUE |
| baseline_vs_XGBoost | LightGBM | XGBoost | PR AUC | 0.9107 | 0.9125 | -0.0017 | -0.0062 to 0.0028 | 0.3816 | 0.3816 | 1.0000 | FALSE | FALSE |
| baseline_vs_XGBoost | RandomForest | XGBoost | PR AUC | 0.8870 | 0.9125 | -0.0255 | -0.0374 to -0.0164 | 0.0020 | 0.0024 | 0.0120 | TRUE | TRUE |
| baseline_vs_XGBoost | LogisticRegression | XGBoost | PR AUC | 0.8869 | 0.9125 | -0.0256 | -0.0418 to -0.0108 | 0.0020 | 0.0024 | 0.0120 | TRUE | TRUE |
| baseline_vs_XGBoost | SVM | XGBoost | PR AUC | 0.8750 | 0.9125 | -0.0375 | -0.0529 to -0.0237 | 0.0020 | 0.0024 | 0.0120 | TRUE | TRUE |
| baseline_vs_XGBoost | KNN | XGBoost | PR AUC | 0.6662 | 0.9125 | -0.2462 | -0.2877 to -0.2070 | 0.0020 | 0.0024 | 0.0120 | TRUE | TRUE |
| baseline_vs_XGBoost | GBDT | XGBoost | PR AUC | 0.8919 | 0.9125 | -0.0205 | -0.0307 to -0.0118 | 0.0020 | 0.0024 | 0.0120 | TRUE | TRUE |
| sampling_vs_unsampled_base | ENN-BLSMOTE-XGBoost | XGBoost | PR AUC | 0.9747 | 0.9774 | -0.0027 | -0.0046 to -0.0008 | 0.0060 | 0.0300 | 0.0599 | TRUE | FALSE |
| sampling_vs_unsampled_base | ENN-BLSMOTE-XGBoost | XGBoost | PR AUC | 0.9003 | 0.9125 | -0.0121 | -0.0203 to -0.0047 | 0.0020 | 0.0100 | 0.0200 | TRUE | TRUE |
| XGBoost_sampling_operating_point | ENN-BLSMOTE-XGBoost | XGBoost | Sensitivity | 0.8478 | 0.7397 | 0.1081 | 0.0857 to 0.1331 | 0.0020 | 0.0026 | 0.0360 | TRUE | TRUE |
| XGBoost_sampling_operating_point | ENN-BLSMOTE-XGBoost | XGBoost | PPV | 0.7476 | 0.8694 | -0.1218 | -0.1459 to -0.1012 | 0.0020 | 0.0028 | 0.0360 | TRUE | TRUE |
| XGBoost_sampling_operating_point | ENN-BLSMOTE-XGBoost | XGBoost | Specificity | 0.9404 | 0.9769 | -0.0365 | -0.0426 to -0.0313 | 0.0020 | 0.0026 | 0.0360 | TRUE | TRUE |
| XGBoost_sampling_operating_point | ENN-BLSMOTE-XGBoost | XGBoost | F1 | 0.7946 | 0.7994 | -0.0048 | -0.0238 to 0.0130 | 0.5954 | 0.6721 | 1.0000 | FALSE | FALSE |
| XGBoost_sampling_operating_point | ENN-BLSMOTE-XGBoost | XGBoost | F2 | 0.8257 | 0.7625 | 0.0632 | 0.0433 to 0.0841 | 0.0020 | 0.0028 | 0.0360 | TRUE | TRUE |
| XGBoost_sampling_operating_point | ENN-BLSMOTE-XGBoost | XGBoost | VME | 0.1522 | 0.2603 | -0.1081 | -0.1331 to -0.0857 | 0.0020 | 0.0026 | 0.0360 | TRUE | TRUE |
| XGBoost_sampling_operating_point | ENN-BLSMOTE-XGBoost | XGBoost | ME | 0.0596 | 0.0231 | 0.0365 | 0.0313 to 0.0426 | 0.0020 | 0.0026 | 0.0360 | TRUE | TRUE |
| XGBoost_sampling_operating_point | ENN-BLSMOTE-XGBoost | XGBoost | Brier | 0.0535 | 0.0452 | 0.0083 | 0.0053 to 0.0114 | 0.0020 | 0.0090 | 0.0360 | TRUE | TRUE |

SMOTE, Synthetic Minority Oversampling Technique; BLSMOTE, Borderline Synthetic Minority Oversampling Technique; ADASYN, Adaptive Synthetic Sampling; TL, Tomek Links; ENN, Edited Nearest Neighbors; NCL, Neighborhood Cleaning Rule; NM1, Neighborhood Majority 1; XGBoost, eXtreme Gradient Boosting; LightGBM, Light Gradient Boosting Machine; RF, Random Forest.

# Supplementary Table S6. Top 15 Feature Mean SHAP Values of ENN-BLSMOTE-XGBoost.

| Feature | Mean SHAP |
| --- | --- |
| ICU Admission | 1.228 |
| Vascular System Disease | 0.879 |
| Days of Indwelling Urinary Catheterization | 0.871 |
| Days of Carbapenems Use | 0.862 |
| Days of Endotracheal Intubation | 0.794 |
| Respiratory System Disease | 0.694 |
| Metabolic Abnormality | 0.648 |
| Respiratory Tract Infection | 0.592 |
| Days of β-Lactamase Inhibitor Combinations Use | 0.523 |
| Days of Quinolones Use | 0.503 |
| Digestive System Disease | 0.482 |
| Nervous System Disease | 0.445 |
| Albumin | 0.400 |
| Urinary System Disease | 0.392 |
| Electrolyte Disturbance | 0.380 |

# Supplementary Table S7. Top 15 Feature Mean SHAP Values of SMOTE-TL-XGBoost.

| Feature | Mean SHAP |
| --- | --- |
| ICU Admission | 1.088 |
| Vascular System Disease | 0.901 |
| Days of Indwelling Urinary Catheterization | 0.859 |
| Days of Carbapenems Use | 0.807 |
| Metabolic Abnormality | 0.694 |
| Respiratory System Disease | 0.620 |
| Days of Endotracheal Intubation | 0.595 |
| Respiratory Tract Infection | 0.544 |
| Days of β-Lactamase Inhibitor Combinations Use | 0.482 |
| Digestive System Disease | 0.472 |
| Days of Quinolones Use | 0.422 |
| Nervous System Disease | 0.390 |
| Albumin | 0.385 |
| Urinary System Disease | 0.382 |
| Electrolyte Disturbance | 0.313 |

# Supplementary Table S8. Top 15 Feature Mean SHAP Values of ENN-XGBoost.

| Feature | Mean SHAP |
| --- | --- |
| ICU Admission | 1.071 |
| Days of Indwelling Urinary Catheterization | 0.913 |
| Vascular System Disease | 0.798 |
| Days of Carbapenems Use | 0.774 |
| Days of Endotracheal Intubation | 0.679 |
| Respiratory System Disease | 0.650 |
| Metabolic Abnormality | 0.596 |
| Respiratory Tract Infection | 0.550 |
| Days of β-Lactamase Inhibitor Combinations Use | 0.448 |
| Digestive System Disease | 0.420 |
| Days of Quinolones Use | 0.401 |
| Albumin | 0.396 |
| Urinary System Disease | 0.369 |
| Nervous System Disease | 0.345 |
| Electrolyte Disturbance | 0.344 |

# Supplementary Table S9. Top 15 Feature Mean SHAP Values of BLSMOTE-XGBoost.

| Feature | Mean SHAP |
| --- | --- |
| ICU Admission | 1.060 |
| Days of Indwelling Urinary Catheterization | 0.877 |
| Days of Carbapenems Use | 0.843 |
| Vascular System Disease | 0.843 |
| Days of Endotracheal Intubation | 0.678 |
| Metabolic Abnormality | 0.619 |
| Respiratory System Disease | 0.610 |
| Respiratory Tract Infection | 0.535 |
| Days of β-Lactamase Inhibitor Combinations Use | 0.474 |
| Days of Quinolones Use | 0.451 |
| Digestive System Disease | 0.441 |
| Nervous System Disease | 0.415 |
| Albumin | 0.381 |
| Urinary System Disease | 0.379 |
| Electrolyte Disturbance | 0.320 |

# Supplementary Table S10. Top 15 Feature Mean SHAP Values of XGBoost.

| Feature | Mean SHAP |
| --- | --- |
| ICU Admission | 0.909 |
| Days of Indwelling Urinary Catheterization | 0.873 |
| Vascular System Disease | 0.769 |
| Days of Carbapenems Use | 0.725 |
| Days of Endotracheal Intubation | 0.596 |
| Metabolic Abnormality | 0.568 |
| Respiratory System Disease | 0.545 |
| Respiratory Tract Infection | 0.508 |
| Days of β-Lactamase Inhibitor Combinations Use | 0.424 |
| Digestive System Disease | 0.396 |
| Albumin | 0.377 |
| Days of Quinolones Use | 0.366 |
| Nervous System Disease | 0.330 |
| Urinary System Disease | 0.324 |
| Electrolyte Disturbance | 0.291 |

# Supplementary Table S11. ICU and Non-ICU Cohort Compositions.

| Subgroup | Records | CR | CS | CR prevalence |
| --- | --- | --- | --- | --- |
| ICU | 2544 | 829 | 1715 | 32.6% |
| non_ICU | 3250 | 170 | 3080 | 5.2% |

# Supplementary Table S12. Performance Metrics of SMOTE-TL-XGBoost for Klebsiella pneumoniae Carbapenem Resistance Prediction Under Varying CR:CS Ratios.

| CR:CS | Prior ROC AUC | Prior PR AUC | Prior Recall | Prior Precision | Prior Specificity | Prior Accuracy | Prior F1 | Prior Brier | Prior Spiegelhalter Z | Prior Spiegelhalter p |
| --- | --- | --- | --- | --- | --- | --- | --- | --- | --- | --- |
| 1:1 | 0.971 (95% CI 0.965–0.977) | 0.970 (95% CI 0.962–0.976) | 0.912 | 0.906 | 0.907 | 0.908 | 0.909 | 0.066 | 8.038 | 0.000 |
| 1:2 | 0.973 (95% CI 0.968–0.977) | 0.947 (95% CI 0.937–0.956) | 0.864 | 0.842 | 0.919 | 0.901 | 0.852 | 0.065 | 10.620 | 0.000 |
| 1:3 | 0.979 (95% CI 0.975–0.982) | 0.943 (95% CI 0.935–0.952) | 0.858 | 0.859 | 0.953 | 0.929 | 0.858 | 0.051 | 4.749 | 0.000 |
| 1:4 | 0.976 (95% CI 0.973–0.980) | 0.917 (95% CI 0.905–0.930) | 0.812 | 0.822 | 0.956 | 0.928 | 0.817 | 0.051 | 8.161 | 0.000 |
| 1:5 | 0.980 (95% CI 0.977–0.983) | 0.917 (95% CI 0.903–0.928) | 0.814 | 0.817 | 0.964 | 0.939 | 0.814 | 0.042 | 5.046 | 0.000 |
| 1:6 | 0.979 (95% CI 0.976–0.982) | 0.899 (95% CI 0.891–0.916) | 0.791 | 0.794 | 0.967 | 0.942 | 0.792 | 0.040 | 4.157 | 0.000 |
| 1:7 | 0.976 (95% CI 0.973–0.979) | 0.876 (95% CI 0.864–0.895) | 0.760 | 0.769 | 0.968 | 0.942 | 0.764 | 0.040 | 6.552 | 0.000 |
| 1:8 | 0.977 (95% CI 0.973–0.980) | 0.876 (95% CI 0.857–0.887) | 0.759 | 0.755 | 0.969 | 0.945 | 0.756 | 0.037 | 4.115 | 0.000 |
| 1:9 | 0.978 (95% CI 0.974–0.980) | 0.871 (95% CI 0.853–0.885) | 0.759 | 0.751 | 0.972 | 0.951 | 0.753 | 0.034 | 1.788 | 0.074 |
| 1:10 | 0.978 (95% CI 0.976–0.981) | 0.854 (95% CI 0.843–0.876) | 0.759 | 0.729 | 0.972 | 0.953 | 0.743 | 0.033 | 4.078 | 0.000 |
| 1:20 | 0.980 (95% CI 0.977–0.982) | 0.805 (95% CI 0.783–0.823) | 0.705 | 0.676 | 0.983 | 0.970 | 0.689 | 0.022 | -5.204 | 0.000 |
| 1:30 | 0.978 (95% CI 0.975–0.981) | 0.750 (95% CI 0.731–0.776) | 0.670 | 0.606 | 0.985 | 0.975 | 0.636 | 0.018 | -10.624 | 0.000 |
| 1:40 | 0.978 (95% CI 0.975–0.980) | 0.714 (95% CI 0.695–0.744) | 0.676 | 0.577 | 0.987 | 0.980 | 0.619 | 0.016 | -18.085 | 0.000 |
| 1:50 | 0.977 (95% CI 0.974–0.980) | 0.689 (95% CI 0.662–0.715) | 0.646 | 0.528 | 0.988 | 0.982 | 0.580 | 0.014 | -20.655 | 0.000 |

# Supplementary Table S13. Performance Metrics of ENN-XGBoost for Klebsiella pneumoniae Carbapenem Resistance Prediction Under Varying CR:CS Ratios.

| CR:CS | Prior ROC AUC | Prior PR AUC | Prior Recall | Prior Precision | Prior Specificity | Prior Accuracy | Prior F1 | Prior Brier | Prior Spiegelhalter Z | Prior Spiegelhalter p |
| --- | --- | --- | --- | --- | --- | --- | --- | --- | --- | --- |
| 1:1 | 0.966 (95% CI 0.952–0.967) | 0.965 (95% CI 0.951–0.968) | 0.842 | 0.915 | 0.911 | 0.881 | 0.871 | 0.088 | 29.254 | 0.000 |
| 1:2 | 0.969 (95% CI 0.963–0.973) | 0.938 (95% CI 0.926–0.948) | 0.927 | 0.789 | 0.879 | 0.894 | 0.850 | 0.078 | 28.159 | 0.000 |
| 1:3 | 0.975 (95% CI 0.970–0.979) | 0.932 (95% CI 0.922–0.944) | 0.888 | 0.798 | 0.925 | 0.916 | 0.840 | 0.061 | 16.770 | 0.000 |
| 1:4 | 0.972 (95% CI 0.970–0.977) | 0.904 (95% CI 0.898–0.924) | 0.858 | 0.765 | 0.935 | 0.920 | 0.808 | 0.057 | 15.701 | 0.000 |
| 1:5 | 0.976 (95% CI 0.973–0.980) | 0.899 (95% CI 0.886–0.916) | 0.836 | 0.763 | 0.949 | 0.931 | 0.797 | 0.049 | 13.552 | 0.000 |
| 1:6 | 0.977 (95% CI 0.974–0.980) | 0.897 (95% CI 0.881–0.910) | 0.811 | 0.766 | 0.958 | 0.937 | 0.788 | 0.043 | 9.495 | 0.000 |
| 1:7 | 0.975 (95% CI 0.972–0.978) | 0.875 (95% CI 0.861–0.892) | 0.784 | 0.755 | 0.964 | 0.942 | 0.768 | 0.041 | 8.004 | 0.000 |
| 1:8 | 0.977 (95% CI 0.972–0.978) | 0.873 (95% CI 0.850–0.882) | 0.756 | 0.744 | 0.966 | 0.942 | 0.743 | 0.039 | 8.701 | 0.000 |
| 1:9 | 0.977 (95% CI 0.974–0.980) | 0.859 (95% CI 0.844–0.877) | 0.753 | 0.740 | 0.971 | 0.949 | 0.746 | 0.035 | 8.362 | 0.000 |
| 1:10 | 0.977 (95% CI 0.974–0.979) | 0.839 (95% CI 0.826–0.861) | 0.734 | 0.727 | 0.973 | 0.951 | 0.729 | 0.034 | 9.309 | 0.000 |
| 1:20 | 0.979 (95% CI 0.976–0.981) | 0.803 (95% CI 0.786–0.825) | 0.666 | 0.771 | 0.990 | 0.975 | 0.711 | 0.019 | -1.467 | 0.142 |
| 1:30 | 0.978 (95% CI 0.976–0.981) | 0.753 (95% CI 0.730–0.776) | 0.615 | 0.728 | 0.992 | 0.980 | 0.665 | 0.015 | -3.293 | 0.001 |
| 1:40 | 0.979 (95% CI 0.977–0.982) | 0.747 (95% CI 0.729–0.773) | 0.629 | 0.757 | 0.995 | 0.986 | 0.685 | 0.011 | -9.808 | 0.000 |
| 1:50 | 0.977 (95% CI 0.974–0.980) | 0.695 (95% CI 0.663–0.714) | 0.590 | 0.697 | 0.995 | 0.987 | 0.638 | 0.011 | -7.489 | 0.000 |

# Supplementary Table S14. Performance Metrics of BLSMOTE-XGBoost for Klebsiella pneumoniae Carbapenem Resistance Prediction Under Varying CR:CS Ratios.

| CR:CS | Prior ROC AUC | Prior PR AUC | Prior Recall | Prior Precision | Prior Specificity | Prior Accuracy | Prior F1 | Prior Brier | Prior Spiegelhalter Z | Prior Spiegelhalter p |
| --- | --- | --- | --- | --- | --- | --- | --- | --- | --- | --- |
| 1:1 | 0.971 (95% CI 0.965–0.977) | 0.969 (95% CI 0.961–0.975) | 0.920 | 0.903 | 0.903 | 0.910 | 0.911 | 0.067 | 7.319 | 0.000 |
| 1:2 | 0.973 (95% CI 0.968–0.977) | 0.945 (95% CI 0.934–0.955) | 0.874 | 0.845 | 0.921 | 0.905 | 0.859 | 0.065 | 10.046 | 0.000 |
| 1:3 | 0.978 (95% CI 0.975–0.982) | 0.940 (95% CI 0.932–0.950) | 0.860 | 0.849 | 0.949 | 0.927 | 0.854 | 0.052 | 5.068 | 0.000 |
| 1:4 | 0.976 (95% CI 0.973–0.980) | 0.918 (95% CI 0.906–0.931) | 0.809 | 0.809 | 0.953 | 0.925 | 0.809 | 0.051 | 8.060 | 0.000 |
| 1:5 | 0.979 (95% CI 0.975–0.982) | 0.914 (95% CI 0.900–0.925) | 0.813 | 0.803 | 0.960 | 0.935 | 0.805 | 0.044 | 6.406 | 0.000 |
| 1:6 | 0.978 (95% CI 0.975–0.981) | 0.895 (95% CI 0.887–0.913) | 0.784 | 0.779 | 0.964 | 0.938 | 0.780 | 0.041 | 5.218 | 0.000 |
| 1:7 | 0.975 (95% CI 0.972–0.979) | 0.869 (95% CI 0.859–0.890) | 0.764 | 0.745 | 0.963 | 0.938 | 0.753 | 0.041 | 8.302 | 0.000 |
| 1:8 | 0.977 (95% CI 0.974–0.980) | 0.875 (95% CI 0.856–0.887) | 0.770 | 0.762 | 0.969 | 0.947 | 0.765 | 0.037 | 5.063 | 0.000 |
| 1:9 | 0.978 (95% CI 0.974–0.980) | 0.868 (95% CI 0.849–0.881) | 0.753 | 0.738 | 0.970 | 0.949 | 0.743 | 0.035 | 3.769 | 0.000 |
| 1:10 | 0.978 (95% CI 0.975–0.981) | 0.855 (95% CI 0.842–0.874) | 0.762 | 0.726 | 0.971 | 0.952 | 0.743 | 0.033 | 4.952 | 0.000 |
| 1:20 | 0.979 (95% CI 0.976–0.982) | 0.806 (95% CI 0.783–0.823) | 0.709 | 0.696 | 0.984 | 0.971 | 0.700 | 0.022 | -4.314 | 0.000 |
| 1:30 | 0.978 (95% CI 0.974–0.980) | 0.745 (95% CI 0.724–0.770) | 0.669 | 0.608 | 0.986 | 0.975 | 0.636 | 0.018 | -6.149 | 0.000 |
| 1:40 | 0.978 (95% CI 0.976–0.981) | 0.713 (95% CI 0.693–0.741) | 0.674 | 0.583 | 0.988 | 0.980 | 0.624 | 0.016 | -11.797 | 0.000 |
| 1:50 | 0.975 (95% CI 0.973–0.978) | 0.682 (95% CI 0.657–0.712) | 0.633 | 0.509 | 0.987 | 0.981 | 0.561 | 0.014 | -14.426 | 0.000 |

# Supplementary Table S15. Performance Metrics of Baseline XGBoost for Klebsiella pneumoniae Carbapenem Resistance Prediction Under Varying CR:CS Ratios.

| CR:CS | Prior ROC AUC | Prior PR AUC | Prior Recall | Prior Precision | Prior Specificity | Prior Accuracy | Prior F1 | Prior Brier | Prior Spiegelhalter Z | Prior Spiegelhalter p |
| --- | --- | --- | --- | --- | --- | --- | --- | --- | --- | --- |
| 1:1 | 0.972 (95% CI 0.966–0.977) | 0.969 (95% CI 0.961–0.976) | 0.919 | 0.902 | 0.902 | 0.909 | 0.910 | 0.066 | 3.058 | 0.127 |
| 1:2 | 0.972 (95% CI 0.967–0.976) | 0.944 (95% CI 0.933–0.953) | 0.862 | 0.862 | 0.931 | 0.908 | 0.861 | 0.066 | 4.198 | 0.176 |
| 1:3 | 0.977 (95% CI 0.974–0.981) | 0.938 (95% CI 0.930–0.949) | 0.824 | 0.862 | 0.956 | 0.923 | 0.843 | 0.053 | 2.444 | 0.162 |
| 1:4 | 0.977 (95% CI 0.974–0.981) | 0.923 (95% CI 0.913–0.937) | 0.789 | 0.843 | 0.963 | 0.929 | 0.815 | 0.048 | 2.510 | 0.189 |
| 1:5 | 0.979 (95% CI 0.975–0.982) | 0.917 (95% CI 0.902–0.927) | 0.786 | 0.848 | 0.972 | 0.940 | 0.814 | 0.042 | 2.205 | 0.188 |
| 1:6 | 0.978 (95% CI 0.975–0.981) | 0.899 (95% CI 0.891–0.916) | 0.754 | 0.839 | 0.977 | 0.946 | 0.794 | 0.039 | 1.935 | 0.265 |
| 1:7 | 0.977 (95% CI 0.973–0.980) | 0.879 (95% CI 0.867–0.896) | 0.726 | 0.825 | 0.978 | 0.947 | 0.771 | 0.038 | 2.802 | 0.129 |
| 1:8 | 0.978 (95% CI 0.975–0.981) | 0.884 (95% CI 0.865–0.895) | 0.733 | 0.818 | 0.979 | 0.952 | 0.772 | 0.034 | 1.931 | 0.094 |
| 1:9 | 0.977 (95% CI 0.973–0.979) | 0.864 (95% CI 0.843–0.877) | 0.690 | 0.799 | 0.981 | 0.952 | 0.738 | 0.034 | 2.864 | 0.112 |
| 1:10 | 0.978 (95% CI 0.975–0.981) | 0.853 (95% CI 0.839–0.872) | 0.706 | 0.791 | 0.981 | 0.956 | 0.745 | 0.031 | 2.445 | 0.195 |
| 1:20 | 0.979 (95% CI 0.976–0.982) | 0.805 (95% CI 0.781–0.821) | 0.639 | 0.798 | 0.991 | 0.975 | 0.707 | 0.019 | 0.694 | 0.370 |
| 1:30 | 0.977 (95% CI 0.974–0.980) | 0.751 (95% CI 0.731–0.777) | 0.630 | 0.768 | 0.993 | 0.982 | 0.691 | 0.015 | -1.236 | 0.318 |
| 1:40 | 0.978 (95% CI 0.976–0.982) | 0.736 (95% CI 0.718–0.765) | 0.628 | 0.735 | 0.994 | 0.986 | 0.677 | 0.012 | -3.754 | 0.005 |
| 1:50 | 0.978 (95% CI 0.976–0.981) | 0.701 (95% CI 0.673–0.725) | 0.586 | 0.663 | 0.994 | 0.986 | 0.619 | 0.011 | -3.503 | 0.096 |

# Supplementary Table S16. Calibration Transportability and Prevalence-Prior Correction Across Five Representative CR:CS Ratios.

| CR:CS ratio | Probability method | ROC AUC (95% CI) | PR AUC (95% CI) | recall | Precision | Specificity | VME | ME | Scaled Brier | Spiegelhalter Z | Spiegelhalter p | Calibration intercept | Calibration slope | ECE |
| --- | --- | --- | --- | --- | --- | --- | --- | --- | --- | --- | --- | --- | --- | --- |
| 1:1 | Raw | 0.974 (0.967–0.980) | 0.972 (0.962–0.981) | 0.854 | 0.932 | 0.938 | 0.146 | 0.062 | 0.705 | 7.342 | 0.000 | 0.823 | 0.787 | 0.051 |
| 1:1 | Locked nested Platt | 0.974 (0.967–0.980) | 0.972 (0.962–0.980) | 0.777 | 0.954 | 0.963 | 0.223 | 0.037 | 0.640 | 4.950 | 0.000 | 1.550 | 1.095 | 0.101 |
| 1:1 | Prevalence-prior-corrected Platt | 0.974 (0.967–0.980) | 0.972 (0.962–0.980) | 0.931 | 0.893 | 0.888 | 0.069 | 0.112 | 0.751 | -1.044 | 0.296 | -0.168 | 1.095 | 0.020 |
| 1:5 | Raw | 0.976 (0.971–0.980) | 0.901 (0.877–0.923) | 0.858 | 0.745 | 0.941 | 0.142 | 0.059 | 0.627 | 9.836 | 0.000 | -0.784 | 0.762 | 0.032 |
| 1:5 | Locked nested Platt | 0.976 (0.971–0.980) | 0.900 (0.876–0.923) | 0.780 | 0.810 | 0.964 | 0.220 | 0.036 | 0.668 | -0.638 | 0.523 | -0.083 | 1.058 | 0.013 |
| 1:5 | Prevalence-prior-corrected Platt | 0.976 (0.971–0.980) | 0.900 (0.876–0.923) | 0.773 | 0.813 | 0.965 | 0.227 | 0.035 | 0.668 | -0.457 | 0.648 | -0.040 | 1.058 | 0.013 |
| 1:10 | Raw | 0.977 (0.971–0.982) | 0.851 (0.810–0.885) | 0.860 | 0.595 | 0.941 | 0.140 | 0.059 | 0.431 | 9.091 | 0.000 | -1.543 | 0.760 | 0.050 |
| 1:10 | Locked nested Platt | 0.977 (0.971–0.982) | 0.849 (0.808–0.884) | 0.787 | 0.683 | 0.963 | 0.213 | 0.037 | 0.565 | -2.908 | 0.004 | -0.846 | 1.054 | 0.031 |
| 1:10 | Prevalence-prior-corrected Platt | 0.977 (0.971–0.982) | 0.849 (0.808–0.884) | 0.689 | 0.793 | 0.982 | 0.311 | 0.018 | 0.616 | -0.898 | 0.369 | -0.070 | 1.054 | 0.007 |
| 1:20 | Raw | 0.975 (0.967–0.982) | 0.777 (0.712–0.832) | 0.858 | 0.422 | 0.941 | 0.142 | 0.059 | 0.007 | 8.955 | 0.000 | -2.227 | 0.765 | 0.061 |
| 1:20 | Locked nested Platt | 0.975 (0.967–0.982) | 0.776 (0.711–0.831) | 0.787 | 0.518 | 0.963 | 0.213 | 0.037 | 0.319 | -4.045 | 0.000 | -1.527 | 1.064 | 0.044 |
| 1:20 | Prevalence-prior-corrected Platt | 0.975 (0.967–0.982) | 0.776 (0.711–0.831) | 0.561 | 0.788 | 0.992 | 0.439 | 0.008 | 0.550 | -1.460 | 0.144 | -0.005 | 1.065 | 0.006 |
| 1:50 | Raw | 0.973 (0.962–0.983) | 0.660 (0.536–0.765) | 0.842 | 0.222 | 0.941 | 0.158 | 0.059 | -1.281 | 8.776 | 0.000 | -3.118 | 0.765 | 0.067 |
| 1:50 | Locked nested Platt | 0.973 (0.961–0.983) | 0.660 (0.535–0.763) | 0.758 | 0.291 | 0.963 | 0.242 | 0.037 | -0.448 | -4.658 | 0.000 | -2.423 | 1.066 | 0.053 |
| 1:50 | Prevalence-prior-corrected Platt | 0.973 (0.961–0.983) | 0.660 (0.535–0.763) | 0.411 | 0.867 | 0.999 | 0.589 | 0.001 | 0.452 | -1.113 | 0.266 | 0.083 | 1.067 | 0.003 |

Raw probabilities are the uncorrected outputs of the resampled ENN–BLSMOTE–XGBoost pipeline. Locked nested-Platt probabilities were generated using the calibration mapping estimated at the natural development prevalence (17.24%). Prevalence-prior-corrected probabilities applied a target-prevalence log-odds offset to the locked calibrated probabilities without fitting a separate ratio-specific Platt model. Classification metrics were calculated at the fixed descriptive threshold of 0.50. VME=FN/(TP+FN)=1−sensitivity; ME=FP/(TN+FP)=1−specificity. The 95% CIs for ROC AUC and PR AUC were obtained from 1,000 patient-cluster bootstrap resamples. Patients, rather than individual records, were sampled with replacement, and all records associated with each sampled patient were retained. Because prevalence-prior correction is monotonic, the ROC AUC, PR AUC, and corresponding CIs are identical to those of the locked nested-Platt probabilities within each ratio. Raw Brier scores are prevalence-dependent and should be interpreted together with scaled Brier, ECE, calibration intercept and slope, and the Spiegelhalter test.

# **Supplementary Fig. S1. Performance Visualization of Hybrid Sampling Pathway A-Optimized Models. (A)Receiver Operating Characteristic (ROC) Curves (with Area Under the Curve (AUC) and 95% Confidence Interval (CI)); (B)Precision-Recall (PR) Curves (with PR AUC and 95% CI); (C)Calibration Curves (with Brier Score and Spiegelhalter's Test Results); (D)Decision Curve Analysis (DCA) for Clinical Net Benefit Assessment. Abbreviations: SMOTE, Synthetic Minority Oversampling Technique; BLSMOTE, Borderline Synthetic Minority Oversampling Technique; TL, Tomek Links; ENN, Edited Nearest Neighbors; XGBoost, eXtreme Gradient Boosting; LightGBM, Light Gradient Boosting Machine; RF, Random Forest.**

# **Supplementary Fig. S2.** **Performance Visualization of Hybrid Sampling Pathway B-Optimized Models. (A)Receiver Operating Characteristic (ROC) Curves (with Area Under the Curve (AUC) and 95% Confidence Interval (CI)); (B)Precision-Recall (PR) Curves (with PR AUC and 95% CI); (C)Calibration Curves (with Brier Score and Spiegelhalter's Test Results); (D)Decision Curve Analysis (DCA) for Clinical Net Benefit Assessment. Abbreviations: SMOTE, Synthetic Minority Oversampling Technique; ADASYN, Adaptive Synthetic Sampling; TL, Tomek Links; NCL, Neighborhood Cleaning Rule; NM1, Neighborhood Majority 1; XGBoost, eXtreme Gradient Boosting; LightGBM, Light Gradient Boosting Machine; RF, Random Forest.**

# Supplementary Fig. S3. **SHAP Summary Dot Plot of SMOTE-TL-XGBoost (Top 15 Features).Abbreviations: ICU, intensive care unit; SHAP, SHapley Additive exPlanations.**

# Supplementary Fig. S4. **SHAP Summary Dot Plot of ENN-XGBoost (Top 15 Features).Abbreviations: ICU, intensive care unit; SHAP, SHapley Additive exPlanations.**

# Supplementary Fig. S5. **SHAP Summary Dot Plot of BLSMOTE-XGBoost (Top 15 Features).Abbreviations: ICU, intensive care unit; SHAP, SHapley Additive exPlanations.**

# Supplementary Fig. S6. **SHAP Summary Dot Plot of XGBoost (Top 15 Features).Abbreviations: ICU, intensive care unit; SHAP, SHapley Additive exPlanations.**

# Supplementary Fig. S7. Performance of ENN-BLSMOTE-XGBoost Under Varying CR:CS Ratios. (A)Receiver Operating Characteristic (ROC) Curves Across CR:CS Ratios (with AUC and 95% Confidence Interval); (B)Precision-Recall (PR) Curves Across CR:CS Ratios (with PR AUC and 95% Confidence Interval); (C)Precision-Recall (PR) Curves Across CR:CS Ratios (with PR AUC and 95% Confidence Interval); (D)Decision Curve Analysis (DCA) Across CR:CS Ratios (for Clinical Net Benefit Assessment). Abbreviations: CR, Carbapenem-Resistant Klebsiella pneumoniae; CS, Carbapenem-Susceptible Klebsiella pneumoniae.

# Supplementary Fig. S8. **Performance of XGBoost Under Varying CR:CS Ratios. (A)Receiver Operating Characteristic (ROC) Curves Across CR:CS Ratios (with AUC and 95% Confidence Interval); (B)Precision-Recall (PR) Curves Across CR:CS Ratios (with PR AUC and 95% Confidence Interval); (C)Precision-Recall (PR) Curves Across CR:CS Ratios (with PR AUC and 95% Confidence Interval); (D)Decision Curve Analysis (DCA) Across CR:CS Ratios (for Clinical Net Benefit Assessment). Abbreviations: CR, Carbapenem-Resistant Klebsiella pneumoniae; CS, Carbapenem-Susceptible Klebsiella pneumoniae.**

# Supplementary Fig. S9. **Performance of SMOTE-TL-XGBoost Under Varying CR:CS Ratios. (A)Receiver Operating Characteristic (ROC) Curves Across CR:CS Ratios (with AUC and 95% Confidence Interval); (B)Precision-Recall (PR) Curves Across CR:CS Ratios (with PR AUC and 95% Confidence Interval).Abbreviations: CR, Carbapenem-Resistant Klebsiella pneumoniae; CS, Carbapenem-Susceptible Klebsiella pneumoniae.**

# Supplementary Fig. S10. **Performance of ENN-XGBoost Under Varying CR:CS Ratios. (A)Receiver Operating Characteristic (ROC) Curves Across CR:CS Ratios (with AUC and 95% Confidence Interval); (B)Precision-Recall (PR) Curves Across CR:CS Ratios (with PR AUC and 95% Confidence Interval).Abbreviations: CR, Carbapenem-Resistant Klebsiella pneumoniae; CS, Carbapenem-Susceptible Klebsiella pneumoniae.**

# Supplementary Fig. S11. **Performance of BLSMOTE-XGBoost Under Varying CR:CS Ratios. (A)Receiver Operating Characteristic (ROC) Curves Across CR:CS Ratios (with AUC and 95% Confidence Interval); (B)Precision-Recall (PR) Curves Across CR:CS Ratios (with PR AUC and 95% Confidence Interval).Abbreviations: CR, Carbapenem-Resistant Klebsiella pneumoniae; CS, Carbapenem-Susceptible Klebsiella pneumoniae.**

# Supplementary Fig. S12. Sequential Forward Selection (SFS) for Feature Optimization in the ENN-BLSMOTE-XGBoost Model.
